# MKK6 controls T3-mediated browning of white adipose tissue

**DOI:** 10.1038/s41467-017-00948-z

**Published:** 2017-10-11

**Authors:** Nuria Matesanz, Edgar Bernardo, Rebeca Acín-Pérez, Elisa Manieri, Sonia Pérez-Sieira, Lourdes Hernández-Cosido, Valle Montalvo-Romeral, Alfonso Mora, Elena Rodríguez, Luis Leiva-Vega, Ana Victoria Lechuga-Vieco, Jesús Ruiz-Cabello, Jorge L. Torres, Maria Crespo-Ruiz, Francisco Centeno, Clara V. Álvarez, Miguel Marcos, Jose Antonio Enríquez, Ruben Nogueiras, Guadalupe Sabio

**Affiliations:** 1 Fundación Centro Nacional de Investigaciones Cardiovasculares Carlos III, Calle Melchor Fernández Almagro, 3, 28029 Madrid, Spain; 2 Centro Nacional de Biotecnología, CSIC, Calle Darwin, 3, 28049 Madrid, Spain; 3 Department of Physiology, CIMUS, University of Santiago de Compostela-Instituto de Investigación Sanitaria, Avda. Barcelona, 15782 Santiago de Compostela, Spain; 4 CIBER Fisiopatología de la Obesidad y Nutrición (CIBERobn), Travesía da Choupana, 15706 Santiago de Compostela, Spain; 5 Department of General Surgery, Bariatric Surgery Unit, University of Salamanca, Paseo de San Vicente, 58, 37007 Salamanca, Spain; 6 CIBER Enfermedades respiratorias (CIBERES), Calle Monforte de Lemos, 3-5, 28029 Madrid, Spain; 7 Universidad Complutense de Madrid, Av. Séneca, 2, 28040 Madrid, Spain; 8 Department of Internal Medicine, University Hospital of Salamanca-IBSAL, Paseo de San Vicente, 58, 37007 Salamanca, Spain; 9 Facultad de Ciencias, University of Extremadura, Grupo GIEN (Grupo de Investigación en Enfermedades Neurodegenerativas), Avda. de Elvas, s/n, 06071 Badajoz, Spain; 10 CIBER Fragilidad y Envejecimiento Saludable (CIBERFES), Calle Monforte de Lemos, 3-5, 28029 Madrid, Spain

## Abstract

Increasing the thermogenic capacity of adipose tissue to enhance organismal energy expenditure is considered a promising therapeutic strategy to combat obesity. Here, we report that expression of the p38 MAPK activator MKK6 is elevated in white adipose tissue of obese individuals. Using knockout animals and shRNA, we show that *Mkk6* deletion increases energy expenditure and thermogenic capacity of white adipose tissue, protecting mice against diet-induced obesity and the development of diabetes. Deletion of *Mkk6* increases T3-stimulated UC﻿P1 expression in adipocytes, thereby increasing their thermogenic capacity. Mechanistically, we demonstrate that, in white adipose tissue, p38 is activated by an alternative pathway involving AMPK, TAK, and TAB. Our results identify MKK6 in adipocytes as a potential therapeutic target to reduce obesity.

## Abstract (web summary)

Brown and beige adipose tissues dissipate heat via uncoupling protein 1 (UCP1). Here the authors show that the stress activated kinase MKK6 acts as a repressor of UCP1 expression, suggesting that its inhibition promotes adipose tissue browning and increases organismal energy expenditure.

## Introduction

The incidence of obesity and associated diseases is increasing worldwide. Defined as an exacerbated increase in body weight associated with fat accumulation, obesity is the consequence of a sustained positive energy balance that occurs when energy intake is higher than energy expenditure. Some pharmacological drugs specifically designed to treat obesity have focussed on reducing mainly food intake; however, this approach has had limited efficacy and is associated with undesired secondary effects. Therefore, new strategies are needed to treat obesity and diabetes^1^.

Brown adipose tissue (BAT) is specialized in the dissipation of energy as heat to protect against hypothermia, in a process known as non-shivering thermogenesis^2, 3^. BAT was thought to disappear shortly after birth; however, positron emission tomography identified metabolically active BAT in adults in defined regions, and scattered within white adipose tissue (WAT), suggesting a possible influence on whole-body energy homeostasis^4–6^. The ability to generate heat (thermogenic capacity) depends on uncoupling protein 1 (UCP1)^7^. UCP1 forms a pore in the inner mitochondrial membrane, through which protons can leak, dissipating the electrochemical proton gradient required for ATP synthesis in the mitochondrial matrix. As a result, ATP synthesis is blunted and the energy is released as heat. Brown adipocyte function is regulated in part by thyroid hormones (TH). T3 promotes mitochondrial biogenesis, induces the expression of UCP1, and increases the activity of brown adipocytes^8, 9^. T3 has also been implicated in the induction of the browning of WAT in humans^10^. Increased UCP1 expression in WAT has been suggested as a mechanism for the prevention of obesity^11^. However, little is known about the molecular mechanism controlling this browning process in WAT.

p38α is activated by low temperatures^12^. Moreover, cell-culture studies with p38 inhibitors have identified p38 kinase as a possible mediator of UCP1 expression in the browning^12, 13^. Besides, the p38 downstream target activating transcription factor 2 (ATF2) induces the expression of peroxisome proliferator-activated receptor gamma (PPARγ) co-activator 1α (PGC-1α), and these two nuclear transcription factors together control the expression of UCP1^12^. The stress-activated protein kinase (SAPK) pathway is composed by two main branches: p38 kinases and the c-Jun N-terminal protein kinases (JNK). There are four p38 isoforms (α, β, γ, and δ) and three JNK isoforms (JNK1, 2, and 3)^14^. The JNK pathway has been extensively studied and is implicated in the development of obesity and insulin resistance^15^. In contrast, the role of p38 kinases in this context has received less attention, and their physiological role remains poorly understood.

In this study, we investigated the role of the upstream p38 activator MAPK kinase 6 (MKK6) during obesity induced by a high-fat diet (HFD). Lack of MKK6 increases the basal expression of UCP1 and promotes T3-mediated induction of UCP1 expression in WAT. Moreover, the browning of WAT and subsequent increased energy expenditure in mice lacking MKK6 protects these animals against HFD-induced obesity. This phenotype depends on T3 signaling: the lack of MKK6 increases the sensitivity of adipose tissue to T3-mediated browning. These results indicate that MKK6 is a central regulator of WAT browning and is a possible target for obesity treatment.

## Results

### Lack of MKK6 induces resistance to diet-induced obesity

**Fig. 1:**
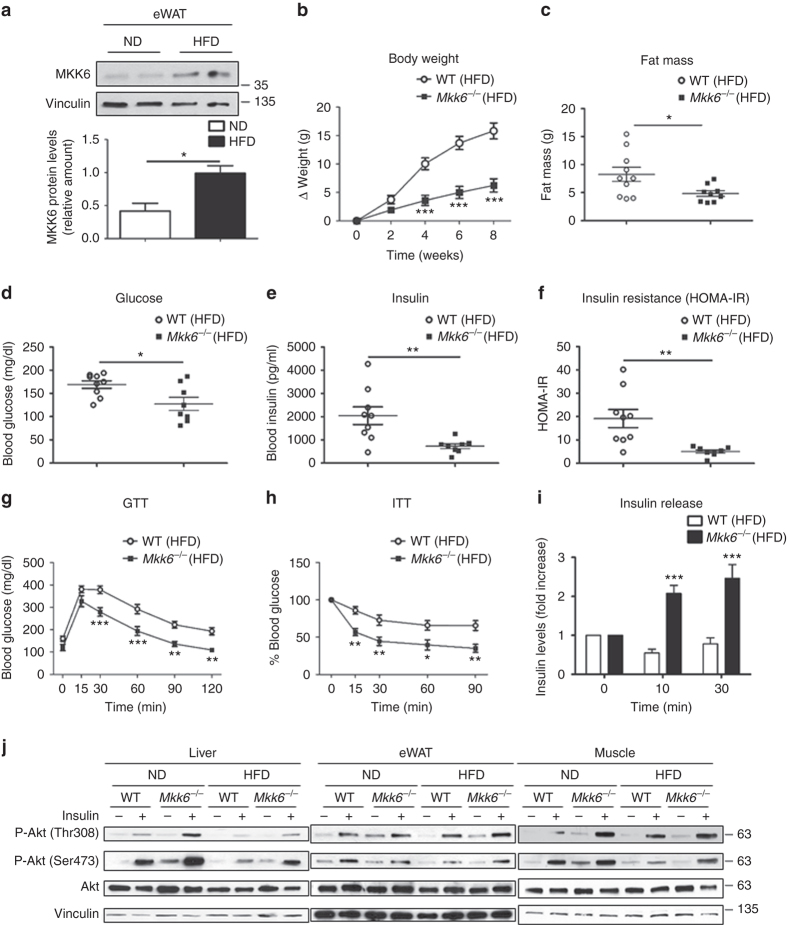
*Mkk6* ^*−/−*^ mice are protected against diet-induced obesity and hyperglycemia **a** Western blot showing elevated MKK6 expression in epididymal white fat (eWAT) from WT mice fed a HFD for 8 weeks. A representative blot from three technical replications (top) and quantification (bottom) are shown (mean ± SEM; *p < 0.05, ND vs HFD *t* test, *n* = 4 mice). **b** Body weight time course in WT and *Mkk6* ^*−/−*^ male mice (8–10 weeks old) fed a HFD over 8 weeks. Data are presented as the increase above initial weight. HFD-induced weight gain was significantly higher in WT than *Mkk6* ^*−/−*^ mice (mean ± SEM, WT *n* = 10 mice; *Mkk6* ^*−/−*^ *n* = 7 mice). **c** Fat mass in *Mkk6* ^*−/−*^ and WT mice after 8 weeks of HFD (mean ± SEM, WT *n* = 10 mice; *Mkk6* ^*−/−*^ *n* = 9 mice). **d**, **e** Fasting blood glucose and insulin in *Mkk6* ^*−/−*^ and WT mice fed the HFD (8 weeks) (mean ± SEM, WT *n* = 9 mice; *Mkk6* ^*−/−*^ *n* = 8 mice). **f** Insulin resistance rate in WT and *Mkk6* ^*−/−*^ mice calculated as homeostasis model assessment (HOMA-IR) ratio (mean ± SEM, WT *n* = 9 mice; *Mkk6* ^*−/−*^ *n* = 8 mice). **g**, **h** Glucose tolerance test (GTT), and insulin tolerance test (ITT) in WT and *Mkk6* ^*−/−*^ mice fed the HFD (8 weeks). Blood glucose concentration was measured in mice given intraperitoneal injections of glucose (1 g/kg) or insulin (0.75 IU/kg) (mean ± SEM, WT *n* = 10 mice; *Mkk6* ^*−/−*^ *n* = 7 mice). **i** Insulin release test in HFD-fed WT and *Mkk6* ^*−/−*^ mice. Mice received i.p. glucose (2 g/kg) after overnight fasting (mean ± SEM, WT *n* = 9 mice; *Mkk6* ^*−/−*^ *n* = 6 mice). **j** Western blot analysis of Akt activation in liver, epididymal white adipose tissue (eWAT), and skeletal muscle from mice fed normal chow diet (ND) or high-fat diet (HFD). Mice were treated without or with insulin (1.5 IU/kg) for 15 min after overnight fasting. Each line represents a pool of tissue from 4 mice.*p < 0.05, **p < 0.01, ***p < 0.001 WT vs *Mkk6* ^*−/−*^ (two-way ANOVA coupled to Bonferroni’s post-tests or *t* test or Welch’s test when variances were different)

MKKs are the activators of the MAPK family members and control multiple cell responses to diverse stimuli^16^. Although certain MKKs and their downstream pathways are known to be activated in human adipose tissue during obesity^17^, the role of MKK6 is still unknown. Analysis of protein levels of MKK6 in fat of lean and obese mice (fed a HFD for 8 weeks) revealed markedly higher levels of MKK6 in epididymal white fat (eWAT) and subcutaneous fat (sWAT) than mice fed a standard chow diet, indicating a possible role of MKK6 in WAT metabolism (Fig. 1a and Supplementary Fig. 1a). No differences were observed in muscle and liver, while BAT presented a reduction in MKK6 expression after HFD (Supplementary Fig. 1a).

To explore this further, we fed HFD to mice lacking MKK6 (*Mkk6* ^*−/−*^). Compared with wild-type (WT) controls, *Mkk6* ^*−/−*^ mice were protected against HFD-induced obesity (Fig. 1b). The lower body weight in *Mkk6* ^*−/−*^ mice correlated with a lower fat mass detected by nuclear magnetic resonance (NMR) (Fig. 1c). Further analysis showed that the weight of liver, eWAT, sWAT, and BAT was lower in *Mkk6* ^*−/−*^ mice (Supplementary Fig. 1b). These differences were associated with smaller adipocytes and lipid droplets (Supplementary Fig. 1c, d) and a protection against liver steatosis (Supplementary Fig. 1c, e).

### *Mkk6*^*−/−*^ mice are protected against diabetes

The reduced fat accumulation in *Mkk6* ^*−/−*^ mice prompted us to investigate whether these mice were protected against HFD-induced diabetes. *Mkk6* ^*−/−*^ mice had significantly lower levels of HFD-induced hyperglycemia and hyperinsulinemia than WT mice, presenting lower insulin resistance (Fig. 1d–f). Moreover, HFD-fed *Mkk6* ^*−/−*^ mice showed enhanced glucose tolerance (Fig. 1g) and insulin sensitivity (Fig. 1h). The greater glucose tolerance in *Mkk6* ^*−/−*^ mice was matched by a higher glucose-induced blood insulin concentration (Fig. 1i). These data indicate that MKK6 deficiency protects against HFD-induced insulin resistance. Western blot analysis showed that suppression of insulin-stimulated AKT activation as a result of HFD was substantially prevented in eWAT from *Mkk6* ^*−/−*^ mice with liver and skeletal muscle also partially protected (Fig. 1j and Supplementary Fig. 1f). These results confirm that HFD-fed *Mkk6* ^*−/−*^ mice have higher systemic insulin sensitivity than WT mice.

### Higher energy expenditure and WAT browning in *Mkk6*^*−/−*^ mice

**Fig. 2:**
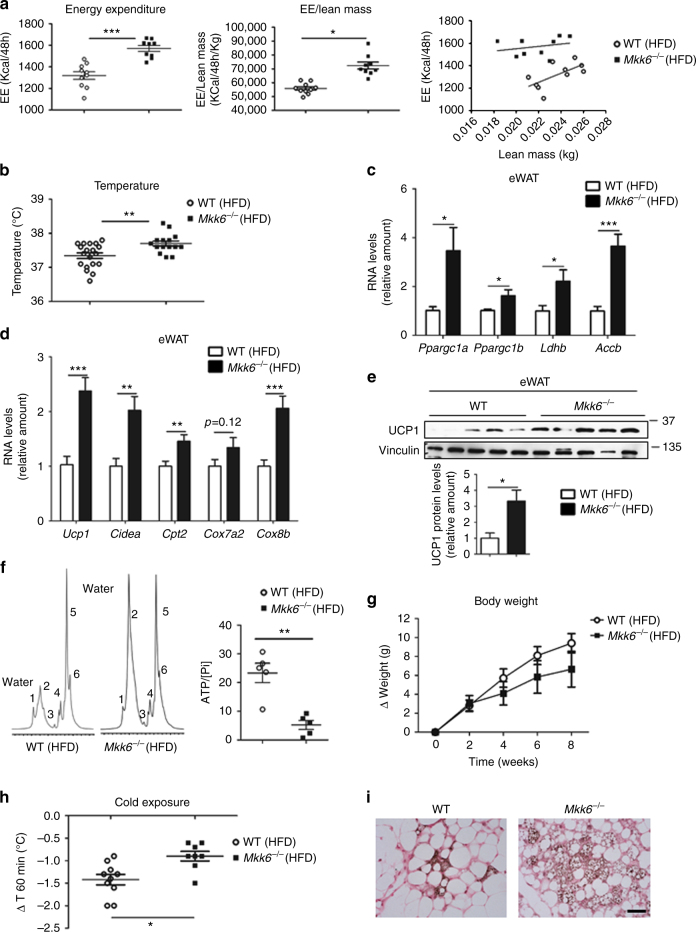
*Mkk6* ^*−/−*^ mice have higher energy expenditure by increased thermogenesis. **a** Comparison of energy balance between HFD-fed WT and *Mkk6* ^*−/−*^ mice. Mice were fed the HFD for 8 weeks and examined in a metabolic cage over a 2-day period to measure energy expenditure (EE). EE levels non-corrected (left), corrected by lean mass (centre), or an ANCOVA analysis (right) are shown (mean ± SEM, WT *n* = 10 mice; *Mkk6* ^*−/−*^ *n* = 9 mice). **b** Body temperature of HFD-fed WT and *Mkk6* ^*−/−*^ mice (mean ± SEM, WT *n* = 19 mice; *Mkk6* ^*−/−*^ *n* = 15 mice). **c**, **d** qRT-PCR analysis of thyroid-hormone-responsive genes and browning-associated genes in epididymal fat (eWAT). Data were normalized to the expression of *Gapdh* mRNA in each sample, and are presented as mean ± SEM (WT *n* = 7–23 mice, *Mkk6* ^*−/−*^ *n* = 5–19 mice). **e** Western blot analysis of uncoupling protein 1 (UCP1) in epididymal (eWAT) of WT and *Mkk6* ^*−/−*^ mice. Quantification of eWAT UCP1 protein levels is also shown (mean ± SEM, *n* = 5 mice). **f** Representative white adipose tissue MR spectrum (from 5 mice) from WT (left) and *Mkk6* ^*−/−*^ (right) mice fed the 8-week HFD. WAT from *Mkk6* ^*−/−*^ shows a BAT-like pattern with two large peaks of emission corresponding to water peak (peak 2 at 4.8 p.p.m.) and lipid component (peak 5 at 1.6–1.3 p.p.m.). Also showed quantification of ATP cellular content respect total (cytosolic and mitochondrial) free inorganic phosphate (Pi) (peak at 5.3–5 p.p.m.) in eWAT (mean ± SEM, *n* = 5 mice). **g** Body weight changes in WT and *Mkk6* ^*−/−*^ mice during the 8-week HFD period; mice were housed at 30 °C. (mean ± SEM, WT *n* = 10 mice; *Mkk6* ^*−/−*^ *n* = 7 mice). **h** Effect of cold exposure (4 °C, 60 min) on body temperature in WT and *Mkk6* ^*−/−*^ mice fed the 8-week HFD (mean ± SEM, WT *n* = 10 mice; *Mkk6* ^*−/−*^ *n* = 8 mice). **i** Staining of UCP1 after 1 week of cold exposure in sWAT. Scale bar: 50 µm. Statistically significant differences between *Mkk6* ^*−/−*^ mice and WT mice are indicated: *p < 0.05; **p < 0.01; ***p< 0.001 (*t* test or Welch’s test when variances were different)

Indirect calorimetry analysis demonstrated that HFD-*Mkk6* ^*−/−*^ mice had higher energy expenditure (EE) that WT independently of its correction or not by lean mass (Fig. 2a) without significant differences in O_2_/CO_2_ gas exchange, locomotor activity, or food intake (Supplementary Fig. 2a). The increased EE by MKK6 deficiency was likely to be a major determinant of MKK6-regulated obesity and pro﻿mpted us to examine thermogenesis. When maintained at room temperature (23 °C), the core body temperature of HFD-fed *Mkk6* ^*−/−*^ mice was higher than their WT counterparts (Fig. 2b). Since BAT is the main regulator of body temperature in mice, we asked whether the lower lipid content in BAT of *Mkk6* ^*−/−*^ mice was caused by thermogenesis due to higher BAT activity. However, analysis with an infrared (IR) camera showed no differences in BAT temperature between genotypes (Supplementary Fig. 2b). Moreover, there were no differences in RNA or protein expression of UCP1, the main enzyme responsible for mitochondria thermogenesis in BAT (Supplementary Fig. 2c). Messenger RNA (mRNA) levels of other genes associated with BAT activity were also unaltered in *Mkk6* ^*−/−*^ mice, including the transcriptional coactivators *Ppargc1a*, *Ppargc1b*, *Cidea*, and the metabolic enzymes *Accb* and *Ldhb* (Supplementary Fig. 2d, e).

Recent reports show that specific stimuli can induce thermogenic capability to WAT in a process called “WAT browning”, in which the adipocytes activate typical genes of BAT^18–20^. Analysis of BAT-associated genes in epididymal and subcutaneous white fat revealed that WAT from *Mkk6* ^*−/−*^ mice expressed elevated mRNA levels of the browning and mitochondrial biogenesis markers as *Cidea* ^21^, and *Cpt1b*, *Cpt2*, and *Cox8b* (Fig. 2c, d and Supplementary Fig. 2g). Moreover eWAT and iWAT showed increased UCP1 at mRNA and protein levels (Fig. 2d, e and Supplementary Fig. 2f). Proton NMR spectroscopy (^1^H-MRS) analysis of the eWAT tissue from HFD-*Mkk6* ^*−/−*^ mice identified a BAT-like spectrum profile (Fig. 2f), with water signal and six fat peaks resolvable in spectroscopy^22^ as is characteristic of BAT (Supplementary Fig. 2h) and different from WAT. Furthermore, in vivo phosphorus NMR spectroscopy (^31^P-MRS) also indicated a lower total ATP content with respect to total free inorganic phosphate (Pi) in *Mkk6* ^*−/−*^ eWAT pointing out to a reduced ATP synthesis (Fig. 2f). On the basis of these observations, we hypothesized that the increased energy metabolism in *Mkk6* ^*−/−*^ mice might be a consequence of increased non-shivering thermogenesis in WAT. To test this, we housed animals at different temperatures. Under thermoneutral conditions (30 °C), when adaptive thermogenesis is not required, weight gain was similar between HFD-fed WT and *Mkk6* ^*−/−*^ mice (Fig. 2g). Besides, HFD-*Mkk6* ^*−/−*^ mice are more resistant to cold exposure (4 °C) (Fig. 2h), and after this browning stimulus, *Mkk6* ^*−/−*^ mice expressed higher UCP1 levels in subcutaneous white fat than their WT counterparts (Fig. 2i). Taken together, these data indicate that the low-weight gain in HFD-fed *Mkk6* ^*−/−*^ mice is due to WAT browning and the associated increase in thermogenesis.

### VMH deletion of *Mkk6* does not affect systemic metabolism

The central nervous system is a key regulator of whole-body metabolism and can control weight gain through several mechanisms including browning^17, 23^. The ventromedial hypothalamus (VMH) is a region that controls feeding and thermoregulation^24^. Thereby, we assessed the metabolic phenotype after the stereotaxic injection of lentivirus containing MKK6 short hairpin RNA (shRNA; sh*Mkk6*) into the VMH. After the injection, mice were fed a HFD for 8 weeks and the levels of MKK6 in the whole hypothalamus were assessed (Supplementary Fig. 3a). Mice treated with sh*MKK6* showed no differences in body weight, body composition, or food intake compared with mice injected with lentivirus containing scramble control shRNA (Supplementary Fig. 3b–e). Furthermore, sh*Mkk6*-treated mice were not protected against HFD-induced glucose intolerance (Supplementary Fig. 3f).

### Peripheral suppression of MKK6 protects against obesity

The above results might suggest that obesity protection in *Mkk6* ^*−/−*^ mice is mediated by a peripheral mechanism, independent of MKK6 signaling in the brain. To investigate this possibility, we administered sh*Mkk6* lentivirus intravenously. Western blot analysis confirmed reduced MKK6 expression in adipose tissue and liver (Supplementary Fig. 4a, b). Intravenous sh*Mkk6* protected mice against HFD-induced obesity (Supplementary Fig. 4c), decreased weight of eWAT, BAT, and liver, but not skeletal muscle (Supplementary Fig. 4d), and gave partial protection against liver steatosis (Supplementary Fig. 4e). In addition, sh*Mkk6*-treated mice had lower fasting serum glucose levels, showing protection against hyperglycemia (Supplementary Fig. 4f). These results thus confirm that the phenotype of *Mkk6* ^*−/−*^ mice has a peripheral origin. To discard a possible role of MKK6 in HFD-induced obesity in muscle and liver, we generated conditional mice of *Mkk6* (Supplementary Fig. 4g). Analysis of mice lacking MKK6 in liver or muscle discarded a role of these organs in the phenotype and suggested a cell autonomus role of MKK6 in WAT (Supplementary Fig. 4i, j).

### p38 activation through the AMPK/TAK/TAB pathway in *Mkk6*^*−/−*^

**Fig. 3:**
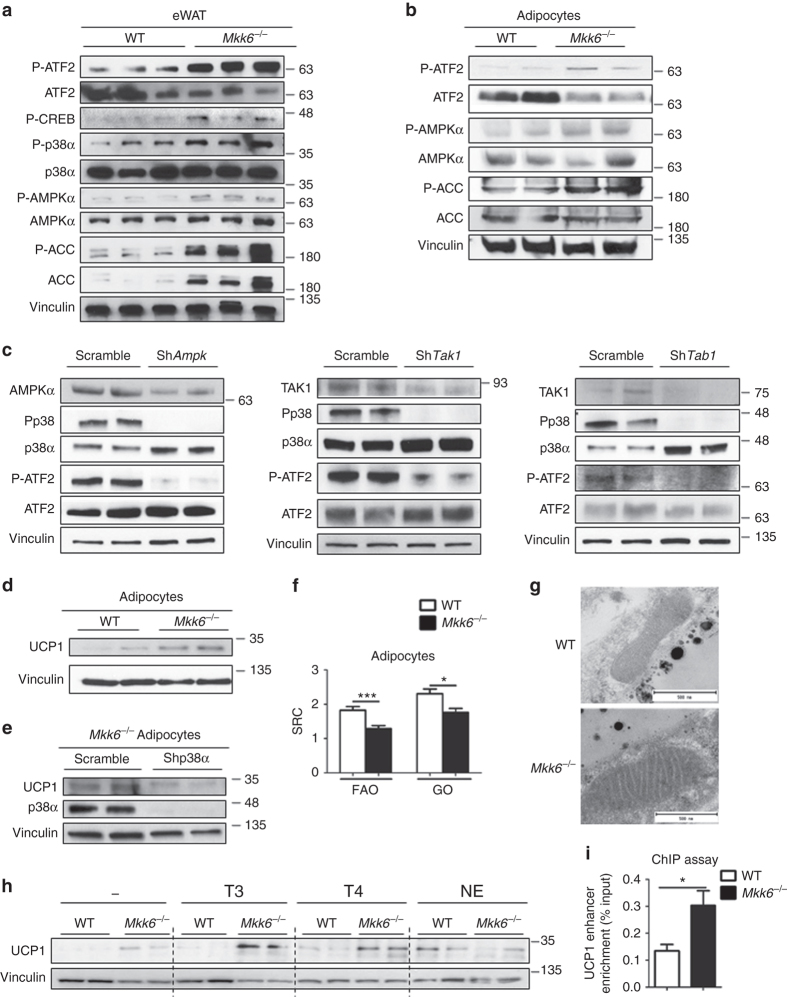
*Mkk6* ^*−/−*^ white adipose tissue is predisposed to T3-induced browning. **a** Activation of p38 and AMPK pathways in epididymal white fat from HFD-fed WT and *Mkk6* ^*−/−*^ mice. Immunoblot analysis with the indicated antibodies was performed in extracts from mice starved overnight. **b** Immunoblot analysis of in vitro differentiated WT and *Mkk6* ^*−/−*^ adipocytes. Representative from three different experiments done by duplicate. **c** *Mkk6* ^*−/*^ pre-adipocytes were infected with shRNA against AMPK, TAK1, TAB1, or scramble as a control. Activation of p38 was assayed by immunoblot of p38 and ATF2 phosphorylation. Representative from three different experiments. **d** UCP1 protein expression in in vitro differentiated WT and *Mkk6* ^*−/−*^ adipocytes. Representative from three different experiments done by duplicate. **e** *Mkk6* ^*−/−*^ pre-adipocytes were infected with shRNA against p38α or scramble as a control, and then differentiated to adipocytes. UCP1 levels were assayed by immunoblot *n* = 4. **f** Mitochondrial spare respiratory capacity (SRC) was assessed by Seahorse assay in primary WT and *Mkk6* ^*−/−*^ adipocytes incubated with glucose oxidation (GO) or fatty acid oxidation (FAO) medium (mean ± SEM, WT *n* = 22 WT or 24 *Mkk6* ^*−/−*^ wells from three mice cultured independently). **g** Representative transmission electronic microscopy images of mitochondria in eWAT from WT and *Mkk6* ^*−/−*^ HFD-fed mice (*n* = 3 mice). Scale bar: 500 nm. **h** Immunoblot analysis of UCP1 protein in in vitro differentiated WT and *Mkk6* ^*−/−*^ adipocytes stimulated for 48 h with T3, T4, or norepinephrine (NE). Representative from three different experiments done by duplicate. **i** Analysis of UCP1 enhancer enrichment in differentiated WAT from WT and *Mkk6* ^*−/−*^ mice after chromatin immunoprecipitation with thyroid hormone receptor α/β antibody. Results are expressed as mean ± SEM (*n* = 12 from three different sets).*p < 0.05, **p < 0.01, ***p < 0.001 WT vs *Mkk6* ^*−/−*^ (*t* test or Welch’s test when variances were different)

BAT expression of UCP1 and PGC1α is believed to be regulated by p38 kinases through activation of the transcription factors ATF2 and CREB^25^. Western blot analysis of WAT from HFD-fed mice demonstrated enhanced activation of p38, ATF2, and CREB in *Mkk6* ^*−/−*^ animals (Fig. 3a). These results are unexpected because MKK6 is a canonical p38 activator. In BAT, GADD45γ induces thermogenic gene expression through activation of p38 after norepinephrin stimulous^26^. To study if lack of MKK6 could affect GADD45γ expression, we performed real-time quantitative reverse transcription PCR (qRT-PCR) analysis of *Gadd45g* expression in eWAT. No differences were found between WT and *Mkk6* ^*−/−*^ animals (Supplementary Fig. 5a). It has been postulated that in absence of the canonical activation, p38 can be triggered by an alternative pathway involving AMPK and TAB1/TAK1 complex^27^. In agreement with this hypothesis, AMPK was hyperactivated in WAT from HFD-fed *Mkk6* ^*−/−*^ mice as judged by phosphorylation levels of AMPK and its substrate ACC (Fig. 3a). Moreover, p38 and AMPK were also hyperactivated in adipocytes derived from *Mkk6* ^*−/−*^ animals as shown by ATF2 and ACC phosphorylation (Fig. 3b). To assess whether AMPK/TAB/TAK1 was involved in p38 hyperactivation, we infected *Mkk6* ^*−/−*^ pre-adipocytes with lentiviral vectors containing shRNA against AMPK, TAB1, and TAK1. Reduction of AMPK, TAK1, or TAB1 protein levels resulted in lower p38 phosphorylation and activation as it is shown by ATF2 phosphorylation (Fig. 3c). These results indicate that in absence of MKK6, adipocytes spontaneously engaged the metabolic master regulator AMPK to trigger p38 activation.

### *Mkk6* deficiency increases adipocyte sensitivity to the T3

**Fig. 4:**
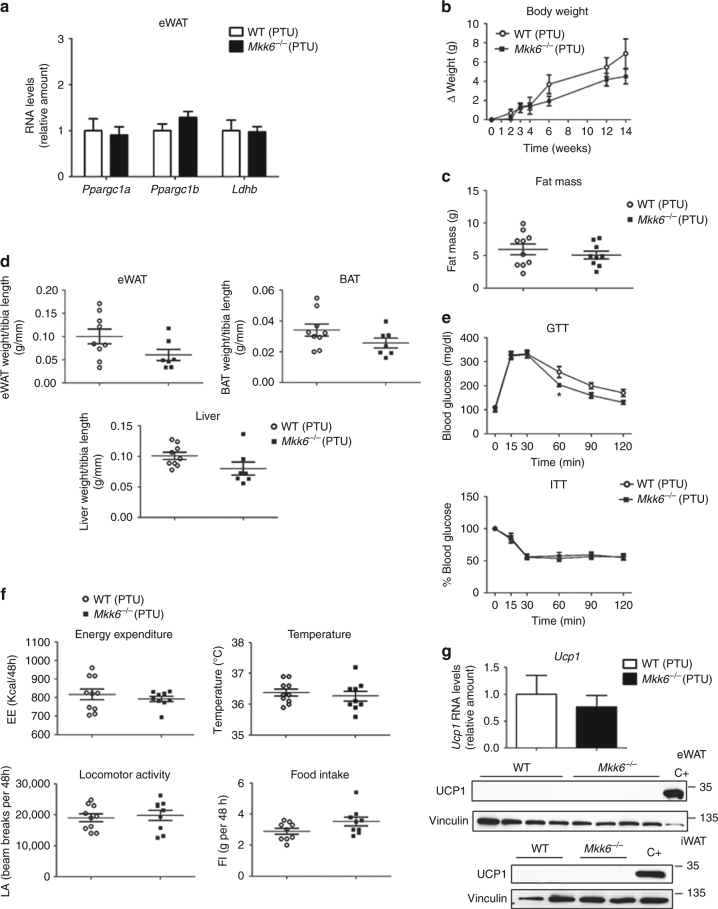
Thyroid hormones participate in the *Mkk6* ^*−/−*^ phenotype. WT and *Mkk6* ^*−/−*^ mice were treated with propylthiouracil (PTU, 1.2 mM), an inhibitor of thyroid hormone synthesis, during the 8-week HFD period. **a** qRT-PCR analysis of genes associated with BAT activity in eWAT. mRNA expression was normalized to the amount of *Gapdh* mRNA (WT *n* = 10 mice; *Mkk6* ^*−/−*^ *n* = 9 mice). **b** Body weight changes in *Mkk6* ^*−/−*^ and WT mice treated with HFD and PTU (WT *n* = 10 mice; *Mkk6* ^*−/−*^ *n* = 9 mice). **c** Fat mass in *Mkk6* ^*−/−*^ and WT mice at the end of the 8-week HFD and PTU treatment (WT *n* = 10 mice; *Mkk6* ^*−/−*^ *n* = 9 mice). **d** Weight of epidydimal white fat (eWAT), brown fat (BAT), and liver relative to tibia length in WT and *Mkk6* ^*−/−*^ mice. (WT *n* = 9 mice; *Mkk6* ^*−/−*^ *n* = 7 mice) **e** Glucose tolerance test (GTT), and insulin tolerance test (ITT) performed in WT and *Mkk6* ^*−/−*^ mice. Overnight or 1-h-starved mice were given an intraperitoneal injection of glucose (1 g/kg) or insulin (0.75 IU/kg), respectively (WT *n* = 9 mice; *Mkk6* ^*−/−*^ *n* = 8 mice). **f** Energy balance at the end of the treatment period, measured in WT and *Mkk6* ^*−/−*^ mice housed in a metabolic cage over 2 days; PTU abolished the enhancement of energy expenditure and body temperature in *Mkk6* ^*−/−*^ mice without changing locomotor activity or food intake. (WT *n* = 10 mice; *Mkk6* ^*−/−*^ *n* = 9 mice except food intake *n* = 9 mice). **g** qRT-PCR analysis of *Ucp1* mRNA (upper panel) and immunoblot analysis of UCP1 protein (lower panels) in eWAT and iWAT. mRNA expression was normalized to the amount of *Gapdh* mRNA (WT *n* = 10 mice; *Mkk6* ^*−/−*^ *n* = 9 mice). Results are expressed as mean ± SEM. *p < 0.05 (two-way ANOVA coupled to Bonferroni’s post-tests or Welch’s test when variances were different)

Next, we addressed whether the effect of MKK6 on WAT function was cell-autonomous using adipocytes from WT and *Mkk6* ^*−/−*^ mice. First, we analyzed whether MKK6 was required to suppress UCP1 expression. In correlation with the increased ATF2 phosphorylation, we found intensified UCP1 levels in *Mkk6* ^*−/−*^ adipocytes (Fig. 3d). These data indicate that lack of MKK6 increased UCP1 expression in adipocytes in a cell-autonomous manner. To asses p38α implication in the increased UCP1 levels detected in *Mkk6* ^*−/−*^ adipocytes, *Mkk6* ^*−/−*^ pre-adipocytes infected with lentiviral vectors containing shRNA against p38α were differentiated to adipocytes and UCP1 levels quantified. Decrease of p38α resulted in a reduction of UCP1 protein levels (Fig. 4e), showing that p38α activation observed in *Mkk6* ^*−/−*^ adipocytes participates in the enhanced UPC1 expression. The higher WAT UCP1 content in *Mkk6* ^*−/−*^ could corroborate substantial mitochondrial proton leak as a mechanism to dissipate energy as heat. To test this possibility, we measured the respiratory capacity of white adipocytes. In concordance with higher UCP1 expression, *Mkk6* ^*−/−*^ adipocytes had a lower spare respiratory capacity than WT adipocytes regardless of the source of the nutrient (glucose or fatty acid) (Fig. 3f). Moreover, transmission electron microscopy analysis of mitochondria from eWAT showed clear differences in mitochondria with higher mitochondria electrodensity observed in the *Mkk6* ^*−/−*^ indicating that this mitochondria are more active than the ones from WT (Fig. 3g). To investigate whether *Mkk6* ^*−/−*^ white pre-adipocytes were more prompted to differentiate to brown adipocytes than WT, white pre-adipocytes were differentiated using a white adipocyte differentiation protocol (WAT) or a brown adipocyte differentiation protocol (WAT + BAT). Only *Mkk6* ^*−/−*^ WAT adipocytes differentiated by the BAT protocol enhanced the oxygen consumption in response to norepinephrine, a characteristic of brown adipocytes, supporting a browning phenotype of *Mkk6* ^*−/−*^ WAT (Supplementary Fig. 5b).

WAT browning in vivo is mediated through a cascade involving β3-adrenergic receptor activation and TH signaling. To investigate which factors contribute to the WAT browning observed in *Mkk6* ^*−/−*^ mice, we monitored UCP1 expression in immortalized *Mkk6* ^*−/−*^ and WT white adipocytes stimulated with T3, T4, and norepinephrine (Fig. 3h). Importantly, adipocytes from *Mkk6* ^*−/−*^ mice were more sensitive to TH stimulation, expressing higher levels of UCP1 than WT cells (Fig. 3h and Supplementary Fig. 5c). *Ucp1* expression is regulated by a highly conserved enhancer element with binding sites for nuclear receptors and bZIP transcriptional factors^28^. Then, we used a chromatin immunoprecipitation (ChIP) assay to analyze the binding of TH receptors (THRs) to the UCP1 enhancer in differentiated WAT from WT and *Mkk6* ^*−/−*^ mice. We observed a significant enrichment in UCP1 enhancer in *Mkk6* ^*−/−*^ adipocytes after ChIP with THRα/β antibody (Fig. 3i) indicating higher sensibility to TH and explaining the increased UCP1 expression. Since our data indicated that p38 activation in WAT was mediated by AMPK, which enhanced UCP1, we evaluated whether T3 could trigger AMPK activation. T3 treatment increased AMPK phosphorylation and activation that correlated with higher levels of UCP1 (Supplementary Fig. 5d).

To determine the relationship between MKK6 loss and T3-induced mitochondrial reorganization, adipocytes were co-stained with the fluorescent dyes Bodipy (which selectively binds to accumulated neutral lipids) and Mitotracker (selective for intracellular mitochondria) (Supplementary Fig. 5e). Lipid droplets looked smaller in *Mkk6* ^*−/−*^ where T3 stimulation seemed to promote lipid droplet breakage that was absent in the WT. The staining correlates with the mitochondrial distribution in T3-treated *Mkk6* ^*−/−*^ indicating enhanced metabolic activity. Together, these data indicate that in *Mkk6* ^*−/−*^ adipocytes T3 promotes a fast lipid utilization by mitochondria in parallel to the increase in UCP1, ultimately leading to the browning of white adipocytes.

### TH contribute to the metabolic actions of MKK6

We next examined the potential causal link thyroid hormone inducing WAT browning and the prevention of HFD-induced weight gain in *Mkk6* ^*−/−*^ mice. Treatment of mice with propylthiouracil (PTU), an inhibitor of thyroid hormone production^29^, suppressed the effects of thyroid hormone-responsive genes in the WAT of *Mkk6* ^*−/−*^ mice (Fig. 4a compared to Fig. 2c, d), the differences in weight and fat mass (Fig. 4b–d), and the changes in glucose and insulin tolerance tests (Fig. 4e) between genotypes. After PTU treatment, EE and body temperature in *Mkk6* ^*−/−*^ mice were similar to readings in WT counterpart, with no changes in locomotor activity or food intake (Fig. 4f). Suppression of TH production also eliminated the increased *Ucp1* expression in WAT (Fig. 4g), while levels in BAT were unaltered (Supplementary Fig. 6a, b). Correct TH inhibition by PTU was assayed by the thyroid expression of genes controlled by TH (Supplementary Fig. 6c). These results indicate that the metabolic phenotype of *Mkk6* ^*−/−*^ mice involves an elevated responsiveness of WAT to TH.

To rule out that part of the effects in vivo were due to changes in the serum TH levels in *Mkk6* ^*−/−*^ mice, we evaluated circulating T3 and T4, and thyroid-stimulating hormone (TSH) levels. While no differences were observed in T3 or T4, serum TSH and pituitary expression of *Tshb* were significantly reduced in *Mkk6* ^*−/−*^ mice (Supplementary Fig. 7a, b) correlating with an increased sensitivity to T3. Although we did not find any change in total hypothalamic content of *Trh* (thyrotropin-releasing hormone) mRNA, or *Ttf1*, *Tshr*, or *Tpo* mRNA in the thyroid gland, we found a substantial repression of thyroglobulin and pendrin (Pds/Slc26A4, the basal iodine transporter), two essential genes in the hormone synthesis pathway in the thyroid, that indicated a physiological extreme downregulation due to T3 hypersensitivity (Supplementary Fig. 7c, d).

We also examined mRNA expression of deiodinases (Dio1, 2, 3) and α and β THRs in eWAT, BAT, and liver. As shown in the Supplementary Fig. 8, the only consistent change in all tissues studied is a reduction in Dio2 expression (the key enzyme converting inactive T4 into active T3) being statistically significant only in BAT. This reduced Dio2 expression would reflect again and a cellular hypersensibility to otherwise normal serum levels of T3.

**Fig. 5:**
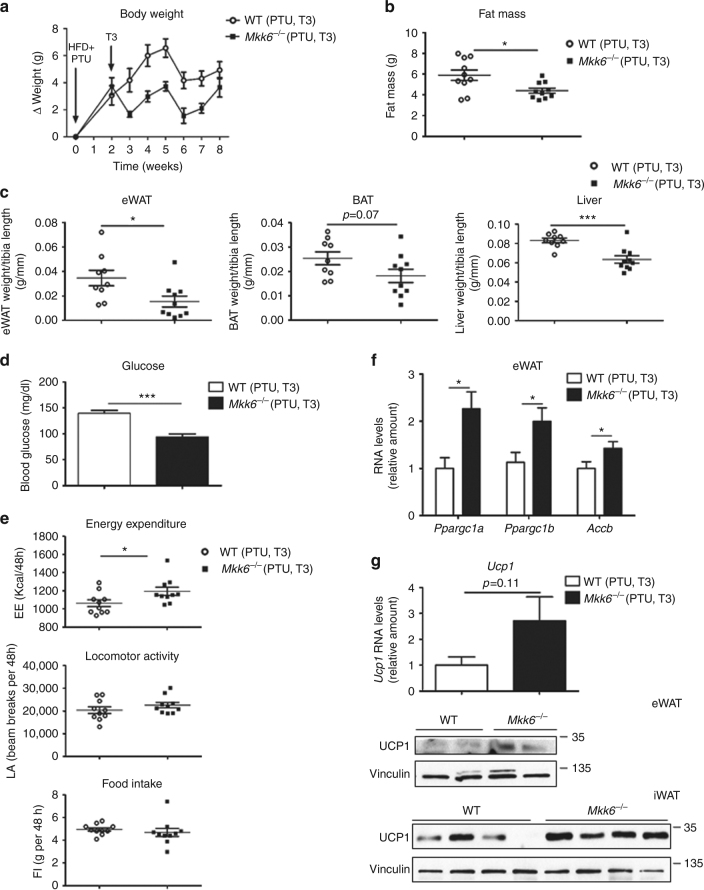
Lack of MKK6 increases peripheral TH sensitivity. WT and *Mkk6* ^*−/−*^ mice were treated with 1.2 mM PTU during the 8-week HFD period. After the first 2 weeks of treatment, mice received daily i.p. injections with T3 (3 μg/100 g in 0.2% BSA–PBS). **a** Effect of T3 on body weight in *Mkk6* ^*−/−*^ and WT mice during the treatment period (*p* < 0.0001 WT vs *Mkk6* ^*−/−*^ two-way ANOVA). **b** Fat mass in *Mkk6* ^*−/−*^ and WT mice at the end of the treatment period. **c** Weight of epididymal white fat (eWAT), brown fat (BAT), and liver relative to tibia length (WT *n* = 9 mice; *Mkk6* ^*−/−*^ *n* = 10 mice). **d** Blood glucose concentration was quantified in mice. **e** Energy expenditure, locomotor activity and food intake determined using metabolic cages. **f** qRT-PCR analysis of genes associated with BAT activity in total RNA extracted from eWAT. mRNA expression was normalized to the amount of *Gapdh* mRNA. **g** qRT-PCR analysis of *Ucp1* mRNA (left) and immunoblot analysis of UCP1 protein (right) in eWAT and iWAT. mRNA expression was normalized to the amount of *Gapdh* mRNA (WT *n* = 9 mice; *Mkk6* ^*−/−*^ *n* = 10 mice except *Ppargc1b* WT *n* = 8 mice, *Mkk6* ^*−/−*^ *n* = 9 mice). Results are expressed as mean ± SEM (*n* = 10 mice, except when is indicated). *p < 0.05, ***p < 0.001 WT vs *Mkk6* ^*−/−*^ (*t* test or Welch’s test when variances were different)

To further investigate TH responsiveness in more detail, we first suppressed endogenous TH production by administering PTU to WT and *Mkk6* ^*−/−*^ mice fed a HFD for 2 weeks, and then treated all animals with T3. Both genotypes showed similar weight gain after treatment with PTU initially (Fig. 5a); however, after addition of exogenous T3 weight gain in *Mkk6* ^*−/−*^ mice decreased with respect to WT mice (Fig. 5a). After 8 weeks of T3 treatment, *Mkk6* ^*−/−*^ mice had lower fat mass and fat accumulation in several tissues than WT mice, (Fig. 5b, c) and improved fed glucose levels (Fig. 5d). T3 treatment also increased EE in *Mkk6* ^*−/−*^ mice (Fig. 5e), correlating with activation of TH-controlled genes in WAT (Fig. 5f, g) with no differences in liver and only increase of *Ppargc1a* in BAT (Supplementary Fig. 9). These results confirm that lack of MKK6 increases the sensitivity of WAT to TH in vivo, resulting in a browning effect in WAT.

### Knocking down *Mkk6* reduces HFD-induced metabolic syndrome

**Fig. 6:**
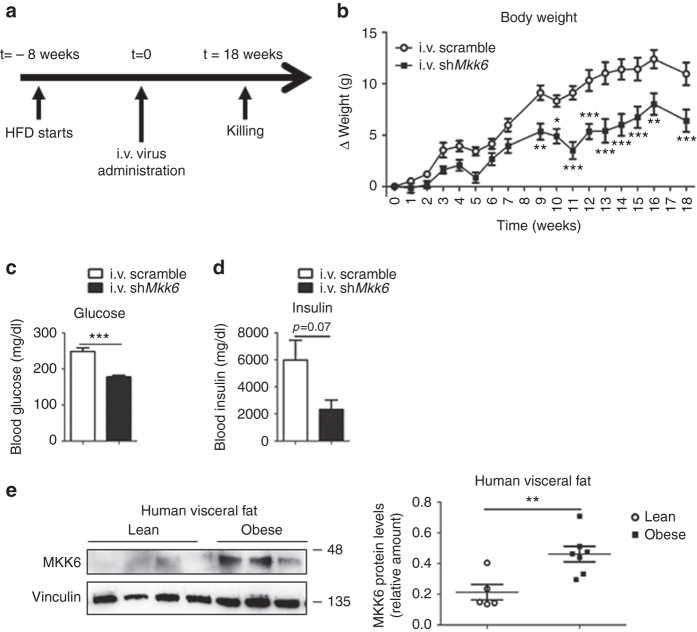
MKK6 depletion has potential as an obesity treatment. **a** WT mice fed a HFD for 8 weeks were injected i.v. with lentivirus containing shRNA against MKK6 or a scramble sequence. **b** Body weight progression in WT mice after injecting lentivirus. Blood glucose (**c**) and insulin (**d**) in scramble or sh*Mkk6*-injected WT mice. Results are expressed as mean ± SEM (*n* = 5 mice). **p < 0.01, ***p < 0.0001 i.v. scramble vs i.v. sh*Mkk6* (two-way ANOVA coupled to Bonferroni’s post-tests or *t* test). **e** Western blot analysis showing elevated MKK6 levels in visceral fat from obese subjects vs controls. Results are expressed as mean ± SEM. **p < 0.01 (lean *n* = 5, obese *n* = 7) A representative blot from three technical replicates (left) and quantification (right) are shown

To test whether depletion of MKK6 has potential as a treatment for obesity, we fed a HFD to WT mice for 8 weeks and then injected them i.v. with lentivirus expressing a scrambled sequence or a shRNA targeting MKK6 (Fig. 6a) that we had already known that reduced MKK6 in adipose tissue (Supplementary Fig. 4a). Mice injected with sh*Mkk6* gained less body mass after lentiviral administration (Fig. 6b) and were protected against HFD-induced hyperinsulinemia and hyperglycemia (Fig. 6c, d). These results indicate that inhibition of MKK6 in peripheral tissues, and particularly in WAT, could have potential in humans as a treatment for obesity and the associated diabetes.

To further study whether these effects observed in mice would be transferable to humans, MKK6 expression in visceral fat from obese and lean individuals was investigated. Analysis of MKK6 protein levels in visceral fat revealed markedly higher levels of MKK6 in obese patients (Fig. 6e).

**Fig. 7:**
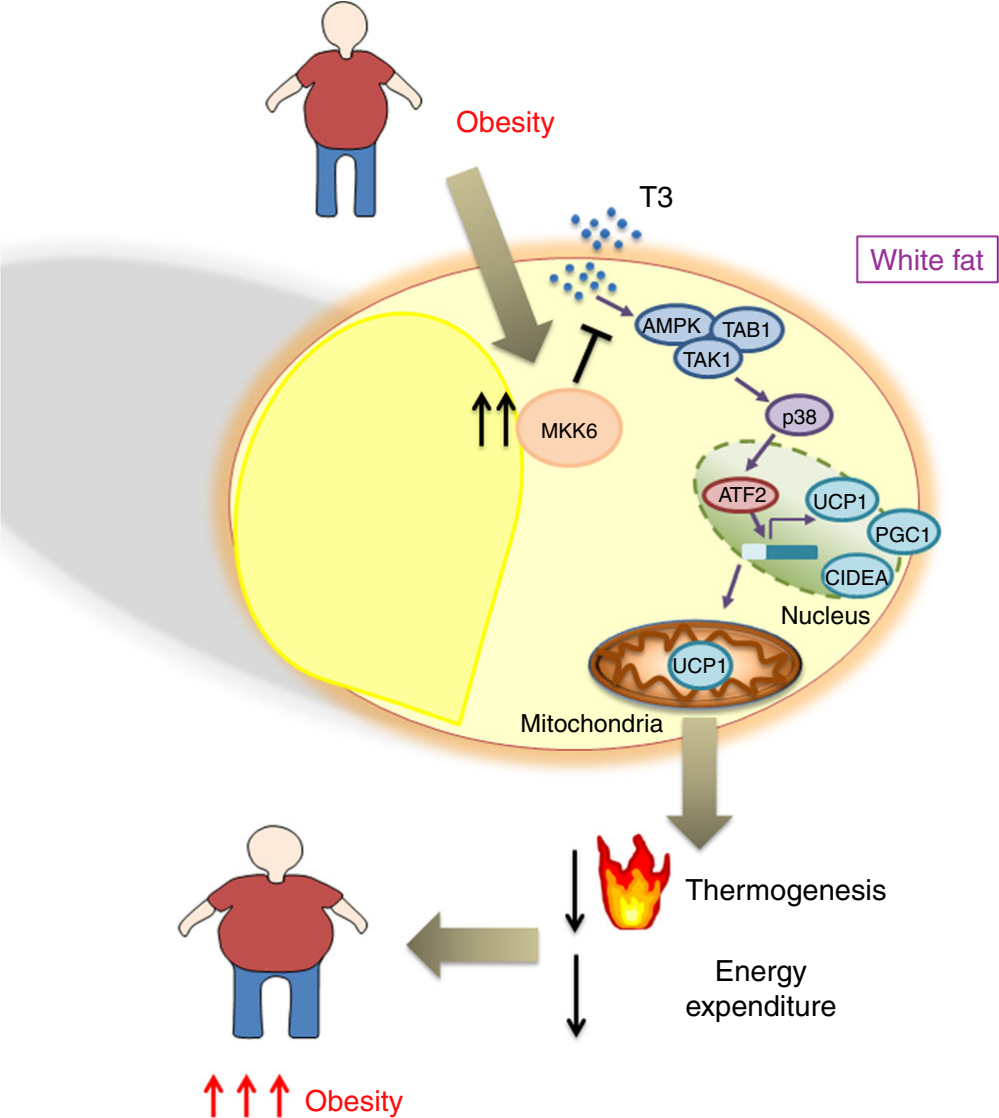
MKK6 is an important regulator of browning. In obesity, high MKK6 levels block UCP1 induction by T3. Reduction in UCP1 levels decreases thermogenesis and energy expenditure. MKK6 could be a therapeutic target to reduce overweight

In conclusion, our data demonstrate that the expression of MKK6 in WAT is important to establish the obese phenotype and resistance to TH. Moreover, its ablation allows WAT UCP1 expression and browning mediated by normal levels of T3 with the consequent increase in EE ameliorating obesity and diabetes (Fig. 7).

## Discussion

In this report, we show that the activity of the mitogen protein kinase MKK6 prevents the browning of WAT. Thus, the WAT of mice lacking this kinase have elevated expression of UCP1, the landmark of browing, as well as other important regulators of this process, such as CIDEA, PGC1α, and PGC1β. Our results demonstrate the physiological relevance of this effect in controlling whole-body metabolism and obesity. Lack of MKK6 increases EE and body temperature, protecting animals from HFD-induced obesity and diet-induced diabetes. WAT browning is required for efficient protection, given that the phenotype is attenuated when animals are kept under thermoneutral conditions, indicating that manifestation of the phenotype requires active thermogenesis. The browning of WAT and the protection against HFD are dependent on the increased sensitivity of *Mkk6* ^*−/−*^ white adipocytes to T3, as they are ablated by inhibition of T3 production with PTU treatment and restored by retreatment with exogenous T3. The role of MKK6 in controlling browning is reinforced by the observation that T3 induces higher levels of UCP1 expression in differentiated *Mkk6* ^*−/−*^ adipocytes than in WT counterparts. Importantly, the results obtained in animal models seem to have clinical relevance, since higher levels of MKK6 were found in the fat of obese patients.

BAT is central in thermogenesis for cold adaptation and energy balance, and depends on the action of UCP1^30^. UCP1 expression is regulated by the sympathetic nervous system, mainly through norepinephrine and thyroid hormone^31^. T3 potentiates the effect of norepinephrine and is essential for the action of UCP1 in cold adaptation^32^. Our observation extends the role of T3 to the control of browning of WAT and the implication of MKK6/p38 in this signaling. Importantly, these effects could be only found in WAT, since the lack of MKK6 in other tissues such as liver or muscle did not cause any relevant metabolic change.

*Mkk6* ^*−/−*^ mice present normal T4 with reduced TSH levels, a whole mark of subclinical hyperthyroidism status^33^. Through different subsets of experiments, we have discarded diferent causes for subclinical hypertiroidism. Our data indicated that hypothalamus was not implicated in the phenotype (Supplementary Figs. 4 and 6). Serum T3 strongly reflects liver production by DIO2^34, 35^. Higher T3 conversion from T4 through increased expression of DIO2 was also discarded (Supplementary Fig. 8). In the other hand, we have obtained relevant data indicating a cellular T3 hypersensitivity in the *Mkk6* ^*−/−*^ mice mainly affecting WAT (Fig. 3). Furthermore, T3 induces stronger UCP1 expression in *Mkk6* ^*−/−*^ adipocytes than in WT (Fig. 3 and Supplementary Fig. 5). In addition, injection of a fixed dose of T3 in PTU hypothyroid mice fed with a HFD recover the lean phenotype (less weight, less hyperglycemia, less fat, and increased browning) only in *Mkk6* ^*−/−*^ mice but not in the WT (Fig. 5). Overall, we have found a positive modulator of T3 action at the white adipocyte cellular level in MKK6 with potential clinical relevance (Fig. 7). Although the implication of TH in metabolic and cellular actions on fat and other tissues including browning have been reported^36, 37^, our data present for the first time an “intracellular modulation of the T3 activity” independently of the THRs, Trh, Tsh, or T4, and with normal levels of T3. These regulation might allow the modulation of T3 action specifically in WAT through MKK6 antagonists, which would prevent the appearance of TH-associated off-target effects in other tissues. These findings have translational relevance because of high proportion of WAT in obese humans and the fact that human beige/brite adipocytes seem to be functional.

We also demonstrate that in WAT, the activation of p38 could be mediated by the alternative mechanism involving AMPK/TAK/TAB. The regulation of p38 activation by AMPK and its interaction with the scaffold protein TAB1 has been shown previously in cardiomiocytes and T cells^27, 38^. However, it was thought that this alternative activation was specific of these tissues. Here, for the first time we show that AMPK/TAB1 pathway controls p38 MAPK activation in white adipocytes. These results have important clinical relevance because activators of AMPK could be used to activate the p38 pathway in adipocytes, and increase browning of WAT through the ATF2 phosphorylation and UCP1 transcription.

In summary, as in obese individuals, the ability to induce browning is reduced^6, 39^, our results have potential clinical significance. In humans, it has highlighted the role of beige/brite fat as opposed to real BAT, hence the increase of MKK6 levels in obese individuals suggest that impaired brite function activation may be in part mediated by this excess of MKK6 in adipose tissue. The higher action of MKK6 axes in visceral fat of obese individuals might contribute to make their adipose tissue resistant to T3, what would underlie the observed resistance to WAT browning. This also agrees with the observation that obese patients have significantly elevated TSH with lower T4 levels, as found not only in a population study with >4600 individuals^40^, but also in a smaller but well-controlled study in 581 obese patients excluding diabetes, thyroid, and other endocrine diseases^41^. The high-TSH/low-T4 profile could be due to a peripheral resistance against T3 induced by obesity^42^ and is also significantly correlated with insulin resistance^40^.

In addition, since it has been recently shown that the expression of browning genes in human WAT correlates with serum T4^4^, our findings suggest that the levels of MKK6 in adipose tissue may be of relevance for the weight gain and weight loss seen in hypo and hyperthyroid patients, respectively. However, further studies should assess this aspect in the appropriate cohort of patients.

Our findings establish a role for MKK6 in the regulation of body energy balance through the modulation of WAT browning, with potential implications for the treatment of obesity.

## Methods

### Study population and sample collection

The study population included 58 adults with body mass index (BMI) ≥35 who underwent elective bariatric surgery at the University Hospital of Salamanca. Patients were excluded if they had a history of alcohol abuse or excessive alcohol consumption (>30 g per day in men and >20 g per day in women), chronic hepatitis C or B. Control subjects (*n* = 13) were recruited among patients who underwent laparoscopic cholecystectomy for gallstone disease. The study was approved by the Ethics Committee of the University Hospital of Salamanca and all subjects provided written informed consent to undergo visceral fat biopsy under direct vision during surgery.

Data were collected on demographic information (age, sex, and ethnicity), anthropomorphic measurements (BMI), smoking and alcohol history, coexisting medical conditions, and medication use. Before surgery, fasting venous blood samples were collected for measuring complete cell blood count, total bilirubin, aspartate aminotransferase (AST), alanine AST (ALT), total cholesterol, high-density lipoprotein, low-density lipoprotein, triglycerides, creatinine, glucose, and albumin (Supplementary Table 1).

### Animal models

The use and generation of C57Bl6J WT mice and knockout mice lacking MKK6 kinase in homozygosis (*Mkk6* ^*−/−*^, B6.129-*Map*2*k*6^tm1Flv^) was previously described^43^. All the animals were maintained on a C57BL/6J background (back-crossed 10 generations). Mice with a germ-line mutation in the *Map2k6* gene and LoxP elements inserted into two introns (Map2k6LoxP) were generated after homologous recombination in ES cells. ES cells were electroporated with this vector (Supplementary Fig. 4g) and selected with 200 μg/ml G418 and 2 μM gangcyclovir. Several correctly targeted ES cell clones were identified by southern blot and PCR. These ES cell clones were injected into C57BL/6J blastocysts to create chimeric mice that transmitted the mutated Map2k6 allele through the germ line. The Flp NeoR cassette was excised by crossing these mice with ACTB:FLPe B6;SJL mice, which express a FLP1 recombinase gene under the direction of the human ACTB promoter. These animals were crossed with FVB-Tg(Ckmm-cre)5Khn/J line on the C57BL/6J background (Jackson Laboratory) to generate mice lacking MKK6 in muscle and with B6.Cg-Tg(Alb-cre)21Mgn/J for deletion in hepatocytes. Genotype was confirmed by PCR analysis of genomic DNA. Male mice were fed with a normal chow diet or a HFD (Research Diets Inc.) for 8 weeks ad libidum. PTU treatment was administrated for 8 weeks in the drinking water at 1.2 mM together with Kool Aid^™^ to improve the taste. In some experiments, T3 (3 μg/100 g in 0.2% BSA–PBS) was injected i.p. daily. For temperature experiments, mice were housed at 30 °C for 8 weeks while feeding a HFD in case of thermoneutral analysis, and exposed to 4 °C for 1 h or 1 week after HFD treatment in case of cold adaptation studies. All animal procedures conformed to EU Directive 86/609/EEC and Recommendation 2007/526/EC regarding the protection of animals used for experimental and other scientific purposes, enacted under Spanish law 1201/2005. The procedures have been reviewed by the Institutional Animal Care and Use Committee (IACUC) of Centro Nacional de Investigaciones Cardiovasculares, and approved by Consejeria de Medio Ambiente, Administración Local y Ordenación del Territorio of Comunidad de Madrid.

### Lentiviral vector production and mice infection

Lentiviruses were produced as described^44^. Briefly, transient calcium phosphate cotransfection of HEK-293 cells was done with the pGIZP empty, pGIZP.sh*Mkk6*, pGIZP.sh*Ampk*, pGIZP.sh*Tak1*, pGIZP.sh*Tab1*, or pGIZT.sh*Mapk14* vectors from Thermo scientific together with pΔ8.9 and pVSV-G. The supernatants containing the LV particles were collected 48 and 72 h after removal of the calcium phosphate precipitate, and were centrifuged at 700 × g, 4 °C for 10 min, and concentrated (165×) by ultracentrifugation for 2 h at 121986 g at 4 °C (Ultraclear Tubes, SW28 rotor and Optima L-100 XP Ultracentrifuge; Beckman). Viruses were collected by adding cold sterile PBS and were titrated by quantitative PCR.

Mice were injected in VMH or tail vein with lentiviral particles suspended in PBS. Seven days after infection, mice were fed a HFD diet.

### Cell culture

For obtaining white pre-adipocites, WT and *Mkk*6^*−/−*^ inguinal fat were mechanically and enzymatically disaggregated using type-A collagenase (2 mg/ml collagenase type-A, Roche) at 37 °C. Cell suspension passed through a 70 µm cell strainer (Falcon) to eliminate stroma and debris, and centrifuged at 400 × g for 8 min at RT. Pellet was collected and cells were counted using a CasyTon cell counter. Pre-adipocytes were inmortalized by infection with SV40T-pBABE-neo virus. Cells were differentiated to adipocytes for 9 days in 8% FCS medium supplemented with 5 μg/ml insulin, 25 μg/ml IBMX, 1 μg/ml dexamethasone, and 1 μM troglitazone. Then cultures were incubated with 100 nM T3, 100 nM T4, and 1 μM norepinephrine for 48 h before extraction. Alternatively, pre-adipocytes were differentiated to adipocytes using brown adipocyte differentiation protocol in which cells were induced to brown fat with 20 nM insulin, 1 nM T3, 125 μM indomethacin, 2 μg/ml dexamethasone, and 50 mM IBMX for 48 h, and maintained with 20 nM of insulin, and 1 nM of T3 for 8 days. In some experiments, white pre-adipocytes were infected with lentivirus containing shRNA targeting AMPK, TAK1, TAB1, p38α, or a scrambled sequence, and selected by resistance to puromycin.

Cell cultures used in this paper were tested for mycoplasma infection.

### Analysis of mitochondrial function

Pre-dipocytes were plated and differentiated in gelatin (0.1%)-coated 96 seahorse plates. T3 stimulation was performed 48 h prior to the oxygen consumption analysis. MitoStress oxygen consumption rate (OCR) was assessed in XF medium containing either 25 mM glucose (glucose oxidation medium) or 1 mM palmitate, 2 mM l-glutamine, and 1 mM sodium pyruvate (fatty acid oxidation medium) using a XF-96 extracellular flux analyzers (Seahorse Bioscience, Agilent Technologies), and data normalized by cell number (CyQuant, Invitrogen). Spare respiration capacity (OCR carbonyl cyanide-4-(trifluoromethoxy)phenylhydrazone (FCCP)/OCR basal) and oxygen consumption in response to norepinephrine (NE) (fold increase (FI) NE/basal) was calculated.

### Western blot

Samples were lysated with RIPA buffer containing protease and phosphatase inhibitors (Tris-Hcl 50 mM, pH 7,5; Triton X-100 1%; EDTA 1 mM, pH 8; EGTA 1 mM; NaF 50 mM; β-glycerophosphate-Na 1 mM; sodium pirophosphate 5 mM; orthovanadate-Na 1 mM; sucrose 0.27 M; PMSF 0.1 mM, β-mercaptoethanol 1 mM, aprotinin 10 µg/ml, and leupectin 5 µg/ml). Lysates were separated by SDS-PAGE and incubated in a 1/1000 dilution with antibodies against phospho-Akt (Thr308) antibody (Cell Signalling Technology cat# 9275s), phospho-Akt (Ser473) antibody (Cell Signalling Technology cat# 9271s), Akt antibody (Cell Signalling Technology cat# 9272s), phospho-ATF2 (Thr69/71) antibody (Cell Signalling Technology cat# 9225s), ATF2 (20F1) antibody (Cell Signalling Technology cat# 9226s), phospho-CREB (Ser133) (87G3) antibody (Cell Signalling Technology cat# 9198), phospho-p38 (Thr180/Tyr182) antibody (Cell Signalling Technology cat# 9211s), phospho-AMPKalpha (Thr172) antibody (Cell Signalling Technology cat# 2531s), AMPKalpha (23A3) antibody (Cell Signalling Technology cat# 2603s), phospho-acetyl-CoA carboxylase (Ser79) antibody (Cell Signalling Technology cat# 3661s), acetyl-CoA carboxylase (C83B10) antibody (Cell Signalling Technology cat# 3676s), TAK1 (D94D7) antibody (Cell Signalling Technology cat# 5206s), TAB1 antibody (C-20) (Santa Cruz Biotechnology cat# sc-6053), p38alpha antibody (C-20) (Santa Cruz Biotechnology cat# sc-535), monoclonal anti-vinculin (clone hVIN-1) antibody (Sigma-Aldrich cat# V9131), MKK6 polyclonal antibody (Stressgen Biotechnologies cat# ADI-KAP-MA014-E), or anti-UCP1 antibody (Abcam cat# AB10983) all used at 1:1000, followed by an incubation with a secondary antibody conjugated with horseradish peroxidase (HRP) (1:5000). Reactive bands were detected by chemioluminiscence and quantified by Image J software. Uncropped western blot images are shown in Supplementary Fig. 10.

### Histology staining

Fresh liver, white fat, and brown fat were fixed with formalin 10%, included in paraffin, and cut in 5-mm slides followed by a hematoxylin and eosin staining. Adipocyte size was quantified using Image J software.

Fat droplets were detected by oil red staining (0.7% in propylenglycol) in 8-mm slides included in OCT compound (Tissue-Tek^©^).

### Glucose tolerance test

Starved overnight mice were injected i.p. with 1 g/kg of glucose and blood glucose levels quantified by an Ascensia Breeze 2 glucose meter at 0, 15, 30, 60, 90, and 120 min post injection.

### Insulin tolerance test

Insulin tolerance test was performed injecting i.p. 0.75 IU/kg of insulin at mice starved 1 h and detecting blood glucose levels by a glucometer at different time points post injection (0, 15, 30, 60, 90, and 120) as indicated in the figure.

### Insulin release and measurement

Mice were injected with 2 g/kg of glucose and blood collected by submaxilar puncture at 0, 10, and 30 min after injection. Insulin was quantified in serum by a multiplexed ELISA with a Luminex 200 analyser (Bio-Rad) following manufacture instructions.

### Indirect calorimetry system

EE, respiratory exchange, locomotor activity, and food intake were quantified using the indirect calorimetry system (TSE LabMaster, TSE Systems, Germany) during 2 days.

### Temperature

Body temperature was detected by a rectal thermometer (AZ 8851K/J/T Handheld Digital Thermometer-Single, AZ Instruments Corp., Taiwan).

BAT-adjacent interscapular temperature was quantified by thermographic images using a FLIR^®^ T430sc Infrared Camera (FLIR Systems, Inc., Wilsonville, Oregon) and analyzed through FlirIR software.

### Magnetic resonance imaging and NMR spectroscopy analysis

Fat mass was analyzed by magnetic resonance imaging (whole body composition analyzer; EchoMRI, Houston, TX, USA).

Spectroscopy examinations of WAT were performed in vivo on a 7T preclinical system (Agilent Varian, Palo Alto, USA) equipped with a DD2 console and an active shielded 205/120 gradient insert coil with 130 mT/m maximum strength. Double-tuned circular transmit/receive coil were used for phosphorus/proton (20 mm), placed over the epididymal fat and BAT (Rapid Biomedical GmBH, Rimpar Germany).

Proton NMR spectra were acquired by 128 transients with 2048 complex points with a spectral bandwidth of 10 kHz and a repetition time of 1.2 ms. Spectra were acquired with adiabatic radiofrequency pulses to improve sensitivity and minimize spectral distortions with an Ernst flip angle. Chemical shifts were expressed relative to the water signal (4.7–4.8 p.p.m.) in ^1^H-MRS and phosphocreatine (0 p.p.m.) in ^31^P-MRS. Signals in NMR spectra were determined quantitatively by integration after automatic or manual baseline correction, with fitting of each peak of the spectrum (after phase and baseline correction) to a Lorentzian function using the Mestrenova program (Mestrelab Research, Santiago de Compostea, Spain; released 2015-02-04 version:10.0.1-14719) on a Macintosh computer. An exponential line broadening (3 Hz for proton) was applied before Fourier transformation.

### qRT-PCR

RNA of 1 mg extracted with RNeasy Plus Mini kit (Quiagen^©^) following manufacture instructions, was transcribed to complementary DNA and qRT-PCR performed using Fast Sybr Green probe (Applied Biosystems) and the appropriated primers in the 7900 Fast Real Time thermocycler (Applied Biosystems). Relative mRNA expression was normalized to *Gapdh* mRNA measured in each sample. Alternatively, RT-PCR was performed using Fast TaqMan probe (Applied Biosystems) and the appropriate TaqMan Assay (Applied Biosystems) in the 7900 Fast Real Time thermocycler. Relative mRNA expression was normalized to 18s mRNA measured in each sample or to *Hprt* mRNA in thyroid analysis. Primers and TaqMan Assays used are specified in Supplementary Table 2.

### UCP1 immunostaining and confocal analysis

For UCP1 immunostaining, fresh fat depots were fixed with formalin 10%, included in paraffin, cut in 5-mm slides, and sequentially stained with a UCP1 antibody (1/500, Abcam cat# AB10983), a biotinylated goat anti-rabbit secondary antibody (1/500, Jackson Immuno Research Laboratories), a streptavidin-conjugated ABC complex, and the substrate 3,3′-diaminobenzidene conjugated with horseradish peroxidase (Vector Laboratories cat# PK-6100), followed by brief counterstaining with Nuclear Fast Red hematoxylin (Sigma).

Alternatively, adipocytes were stained with UCP1 primary antibody (1/500, Abcam cat# AB10983) together with a fluorescent goat anti-rabbit secondary antibody (Invitrogen), Bodipy (Invitrogen), and Dapi (Invitrogen) to study UCP1 expression. Images were captured using a Leica SPE confocal microscope (Leica Microsystems, Wetzlar, Germany).

To analyze mitochondria organization, adipocytes were stained with Mito Tracker Deep Red (Invitrogen) and Bodipy (Invitrogen).

### Mitochondria morphology analysis

Fresh fat depots of 1 mm^2^ were fixed with a mix of paraformaldheyde 4% and glutaraldheyde 2% in 0.4 M hepes buffer for 4 h at 4 °C. Once fixed, samples were washed with 0.4 M hepes buffer and analyzed in a transmission electronic microscope (JEOL 1230) associated to a TVIPS CMOS 4K camera. Pictures were obtained at 80 kV.

### Chromatin immunoprecipitation assay

Immortalized white pre-adipocytes from WT and *Mkk6* ^*−/−*^ mice were differentiated to adipocytes for 9 days and processed to extract chromatin according to SimpleChIP^®^ Plus Kit from Cell Signalling. Chromatin was immunoprecipitated with a THRα/THRβ antibody (C3) (ThermoScientific cat# MA1-215), and, after elution and purification, DNA analyzed by qRT-PCR using UCP1 enhancer primers (fw: TCTACAGCGTCACAGAGGGT, rv: TGATTTCTGCTCTTCTGGCA) and control primers against RPL30 intron 2 supplied by SimpleChIP^®^ Plus Kit. Results are expressed as % of input.

### Hormone circulating levels measurement

T3, T4, and TSH were quantified in serum by a multiplexed ELISA with a Luminex 200 analyser (Bio-Rad) following manufacture instructions.

### Statistical analysis

Results are expressed as mean ± SEM. Statistical analysis was evaluated by Student’s *t* test and two-way ANOVA with values of *p* < 0.05 considered significant.

### Data availability

The authors declare that all the data supporting the findings of this study are available within the paper and its Supplementary Information Files, or available from the authors upon reasonable request.

## Electronic supplementary material

Supplementary Information
