# Supplementary material for: MKK6 controls T3-mediated browning of white adipose tissue
Source: Nat Commun. 2017 Oct 11;8:856. doi: 10.1038/s41467-017-00948-z (PMC5636784; doi:10.1038/s41467-017-00948-z)
Supplement: Supplementary file 1 — Supplementary Information [file 41467_2017_948_MOESM1_ESM.pdf]

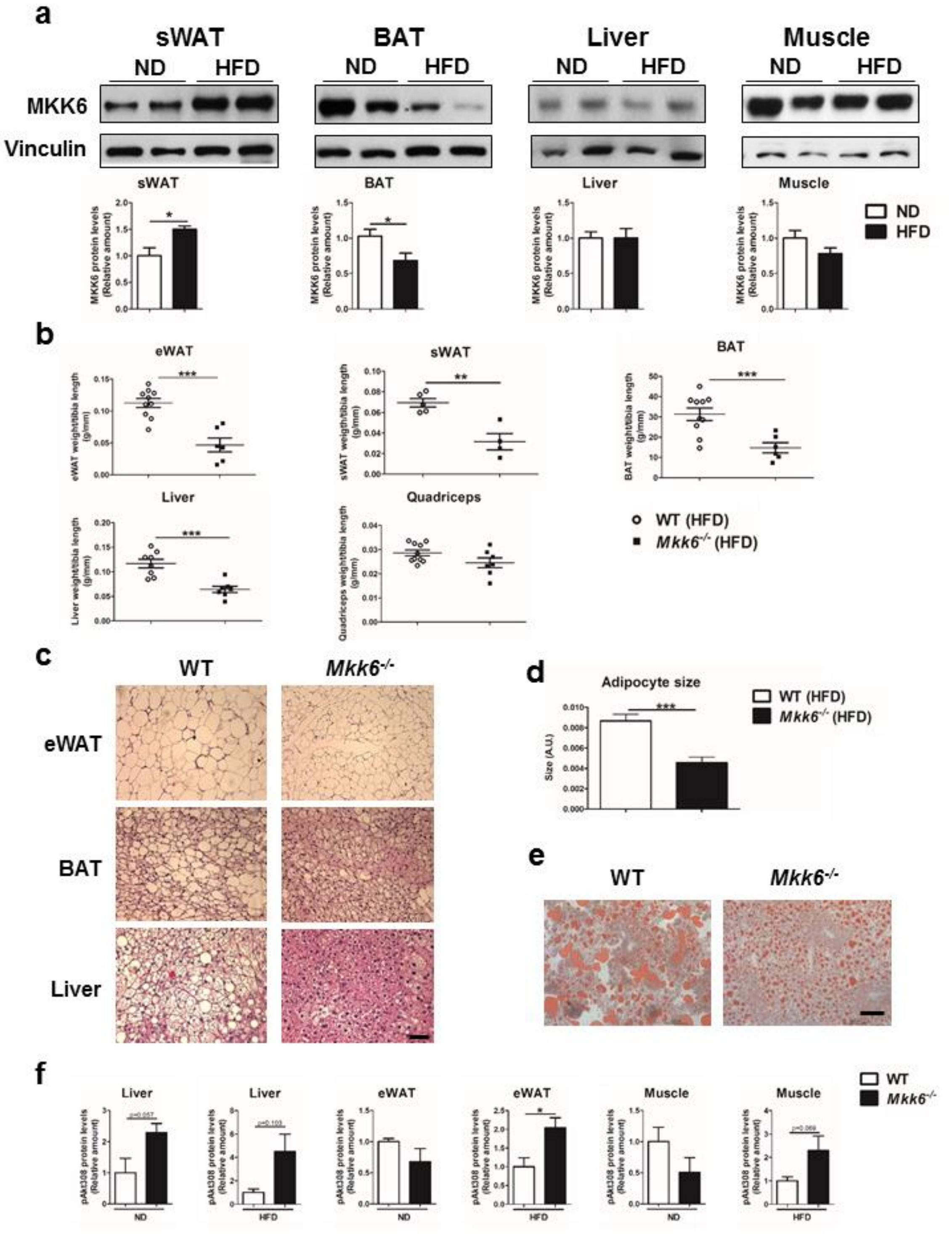

Supplementary Figure 1. Lack of MKK6 confers resistance against diet-induced obesity.

(a) Western blot showing MKK6 expression in subcutaneous fat (sWAT), brown adipose tissue (BAT), liver and skeletal muscle from WT mice fed a normal chow diet (ND) or high-fat diet (HFD). A representative blot (top) and quantification (bottom) are shown. (mean±SEM, sWAT n=5 mice; BAT, liver, muscle ND n=11 mice; HFD n=17 mice). **(b)** WT and *Mkk6*$^{-/-}$ mice were fed a HFD for 8 weeks. Weight of epidydimal white fat (eWAT), sWAT, BAT, liver, and quadriceps with respect to tibia length.

(mean±SEM, eWAT, BAT WT n=10 mice; *Mkk6*$^{-/-}$ n=6 mice; sWAT WT n=5 mice; *Mkk6*$^{-/-}$ n=4 mice; liver WT n=8 mice; *Mkk6*$^{-/-}$ n=7 mice; quadriceps WT n=10 mice; *Mkk6*$^{-/-}$ n=7 mice). **(c)** Representative H&E staining of eWAT, BAT, and liver sections (n= 10 mice (WT) or 7 mice (*Mkk6*$^{-/-}$) and 3 pictures from each mouse). Scale Bar: 50 µm. **(d)** Adipocyte size in eWAT WT and *Mkk6*$^{-/-}$ mice, measured from H&E stained sections using Image J software. (mean±SEM, WT n=9 mice; *Mkk6*$^{-/-}$ n=8 mice) **(e)** Representative oil red O stained liver sections from mice fed the HFD diet (n= 10 mice (WT) or 7 mice (*Mkk6*$^{-/-}$) and 3 pictures from each mouse). Scale Bar: 50µm. **(f)** Quantification of western blots analysing Akt phosphorylation in Thr308 in liver, epidydimal white fat (eWAT), and skeletal muscle from mice fed normal chow diet (ND) or a high-fat diet (HFD). Mice were treated with insulin (1.5 I.U./kg) for 15 min after overnight fasting. Results are expressed as mean±SEM (n=4 mice). *p < 0.05, **p < 0.01, ***p < 0.001 WT vs *Mkk6*$^{-/-}$ (*t*-test or Welch's test when variances were different).

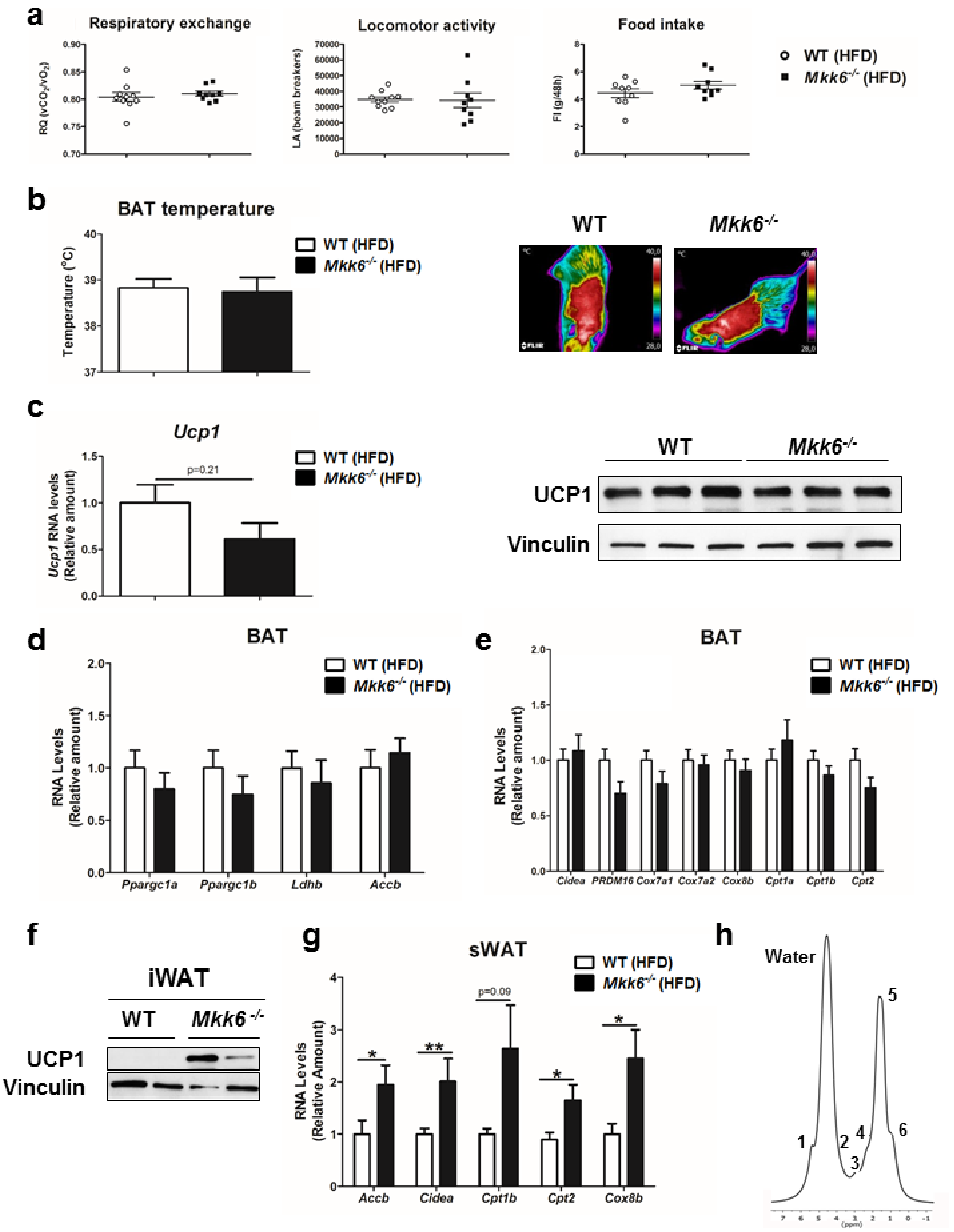

Supplementary Figure 2: Elevated thermogenesis in *Mkk6*$^{-/-}$ mice is not due to activation of BAT.

(a) Comparison of energy balance between HFD-fed WT and *Mkk6*$^{-/-}$ mice. Mice were fed the HFD for 8 weeks and examined in a metabolic cage over a 3-day period to measure respiratory exchange quotient [VCO2]/[VO2], physical activity and food consumption (mean±SEM, WT n=10 mice; *Mkk6*$^{-/-}$ n=9 mice). **(b)** Skin temperature surrounding interscapular brown adipose tissue (BAT) in WT and *Mkk6*$^{-/-}$ mice. Right panels show representative infrared thermal images (mean±SEM n=5 mice). **(c)** qRT-

PCR detection of *Ucp1* mRNA (left) and immunoblot detection of UCP1 protein (right) in BAT. mRNA expression was normalized to the amount of *Gapdh* mRNA. (mean±SEM, WT n=7 mice; *Mkk6*$^{-/-}$ n=4 mice). **(d, e)** qRT-PCR analysis of genes associated with BAT activity or browning in BAT. mRNA expression was normalized to the amount of *Gapdh* mRNA. (mean±SEM, WT n=7/17 mice; *Mkk6*$^{-/-}$ n=4/11 mice). **(f)** Western blot analysis of UCP1 in inguinal fat (iWAT) of WT and *Mkk6*$^{-/-}$ mice. **(g)** qRT-PCR analysis of genes associated with browning in subcutaneous fat (sWAT). mRNA expression was normalized to the amount of *Gapdh* mRNA. (mean±SEM, WT n=10 mice; *Mkk6*$^{-/-}$ n=7 mice). **(h)** Proton Nuclear Magnetic Resonance (NMR) spectroscopy (1H-MRS) analysis of the brown adipose tissue from WT mice. *$p < 0.05$, **$p < 0.01$ WT vs *Mkk6*$^{-/-}$ (*t*-test or Welch's test when variances were different).

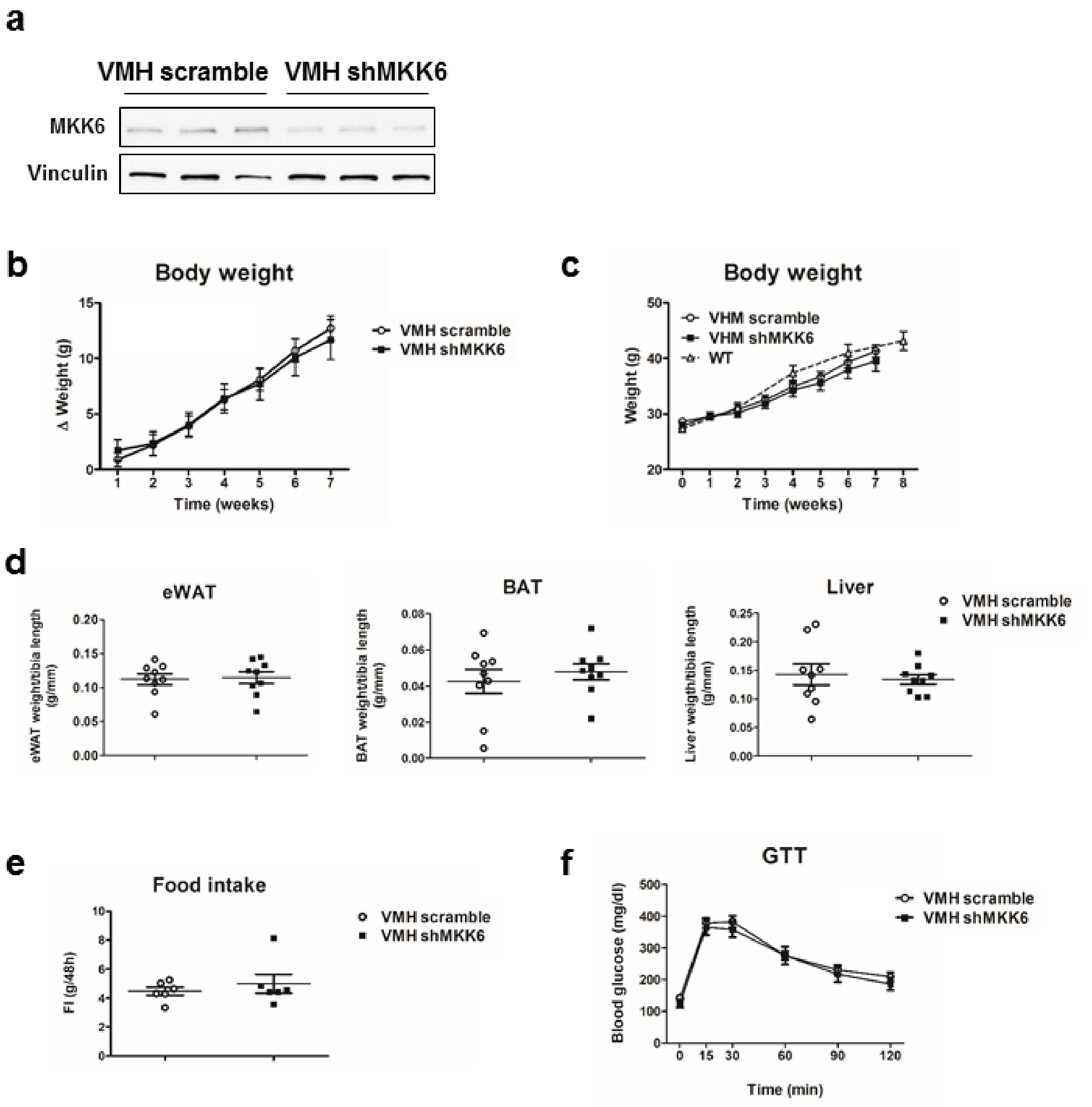

Supplementary Figure 3. Obesity protection in *Mkk6*$^{-/-}$ mice is mediated by a mechanism independent of MKK6 signaling in the brain.

WT mice were administered a lentivirus containing shRNA against *Mkk6* or a scramble sequence by direct stereotaxic injection into the ventromedial hypothalamus (VMH); one week later (week 0), mice were put on the 8-wk HFD. **(a)** Western blot showing *Mkk6* deletion in hypothalamus. **(b)** Body weight gain after 7 weeks on HFD. **(c)** Time course of body weight in injected mice in comparison with WT mice without stereotaxic injection. **(d)** Tissue weight after HFD in CNS-injected mice. **(e)** Food intake during the HFD period in CNS-injected mice. **(f)** Glucose tolerance measured in VMH injected mice at the end of the diet treatment. Blood glucose concentration was measured in mice given an intraperitoneal glucose injection (1 g/kg) after overnight fasting. Results are expressed as mean±SEM (VMH scramble n=9 mice; VMH sh*Mkk6* n=9 mice; WT n=10 mice).

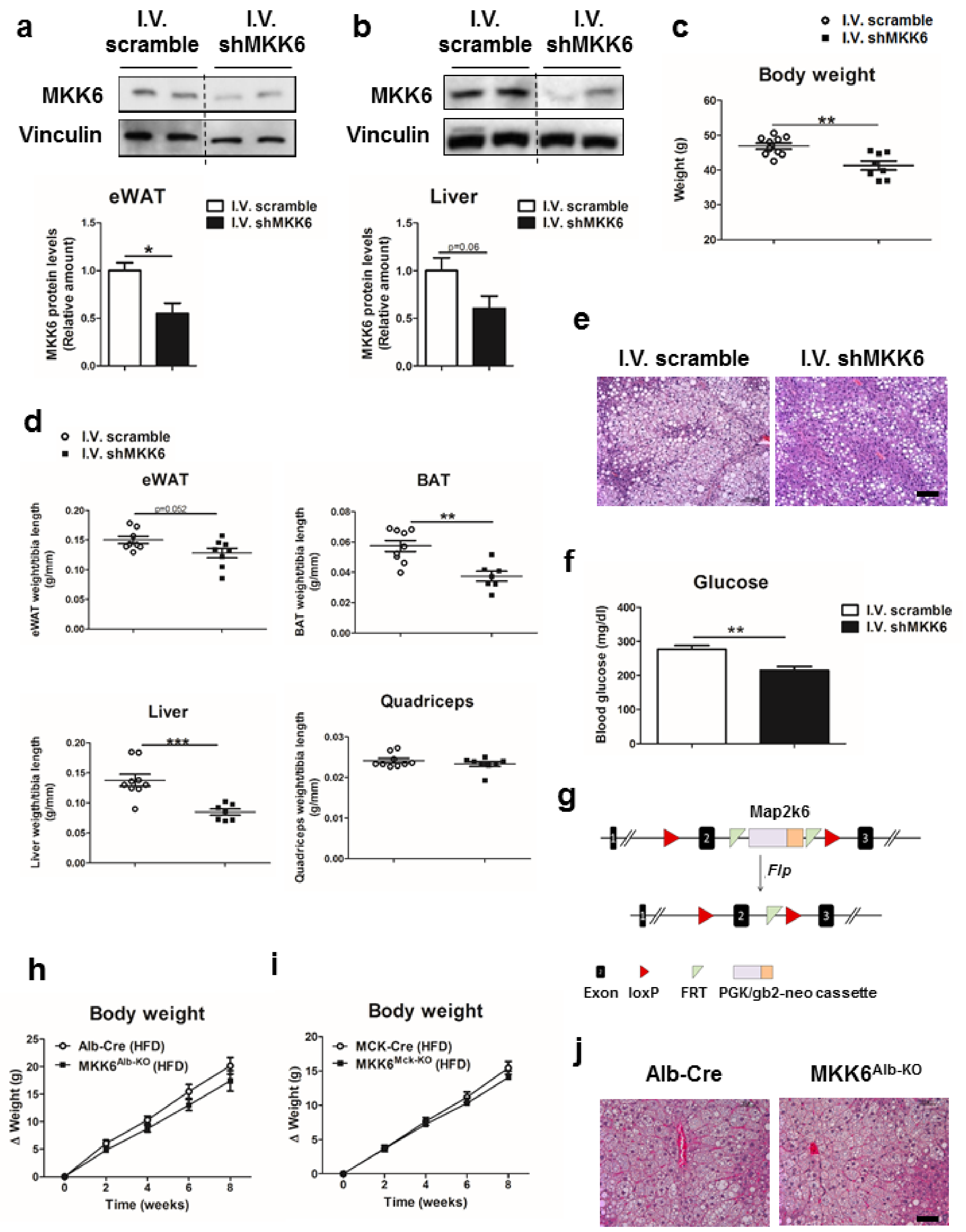

Supplementary Figure 4. Obesity protection in *Mkk6*$^{-/-}$ mice is mediated by a peripheral mechanism.

WT mice were administered with lentivirus containing shRNA against *Mkk6* or a scramble sequence by intravenous injection; one week later (week 0), mice were put on the 8-wk HFD. **(a, b)** Western blot and quantification showing MKK6 deletion in eWAT **(a)** and liver **(b)** after intravenous shMKK6 injection and 8-wk HFD. (mean±SEM, eWAT: WT n=6 mice; *Mkk6*$^{-/-}$ n=9 mice; liver n=5 mice) **(c, d)** Body weight and tissue weight after HFD in i.v. injected mice. (mean±SEM, WT n=10 mice;

Mkk6$^{-/-}$ n=8 mice) **(e)** Representative H&E stained liver sections from i.v. injected mice fed the HFD. (n= 10 mice (WT) or 8 mice (*Mkk6*$^{-/-}$) and 3 pictures from each mouse). Scale Bar: 50 µm. **(f)** Blood glucose in i.v. injected animals after HFD. (mean±SEM, WT n=10 mice; *Mkk6*$^{-/-}$ n=9 mice) **(g)** Cartoon showing the strategy in the generation of MKK6 conditional knockout mice. **p < 0.01, ***p < 0.001 I.V. scramble vs I.V. shMKK6 (*t*-test). **(h, i)** Body weight time course in male mice lacking *Mkk6* in liver (MKK6$^{Alb\text{-}KO}$) and in muscle (MKK6$^{MCK\text{-}KO}$) and the corresponding controls (Alb-Cre, and MCK-Cre, respectively) fed a HFD over 8 weeks. No significant differences were found. Results are expressed as mean±SEM (Alb-Cre n=8 mice; MKK6$^{Alb\text{-}KO}$ n=6 mice; MCK-Cre n=10 mice MKK6^{MCK} n=11 mice). **(j)** Representative H&E stained liver sections from mice fed the HFD (n= 8 mice (Alb-Cre) or 6 mice (MKK6$^{Alb\text{-}KO}$) and 3 pictures from each mouse). Scale Bar: 50 µm.

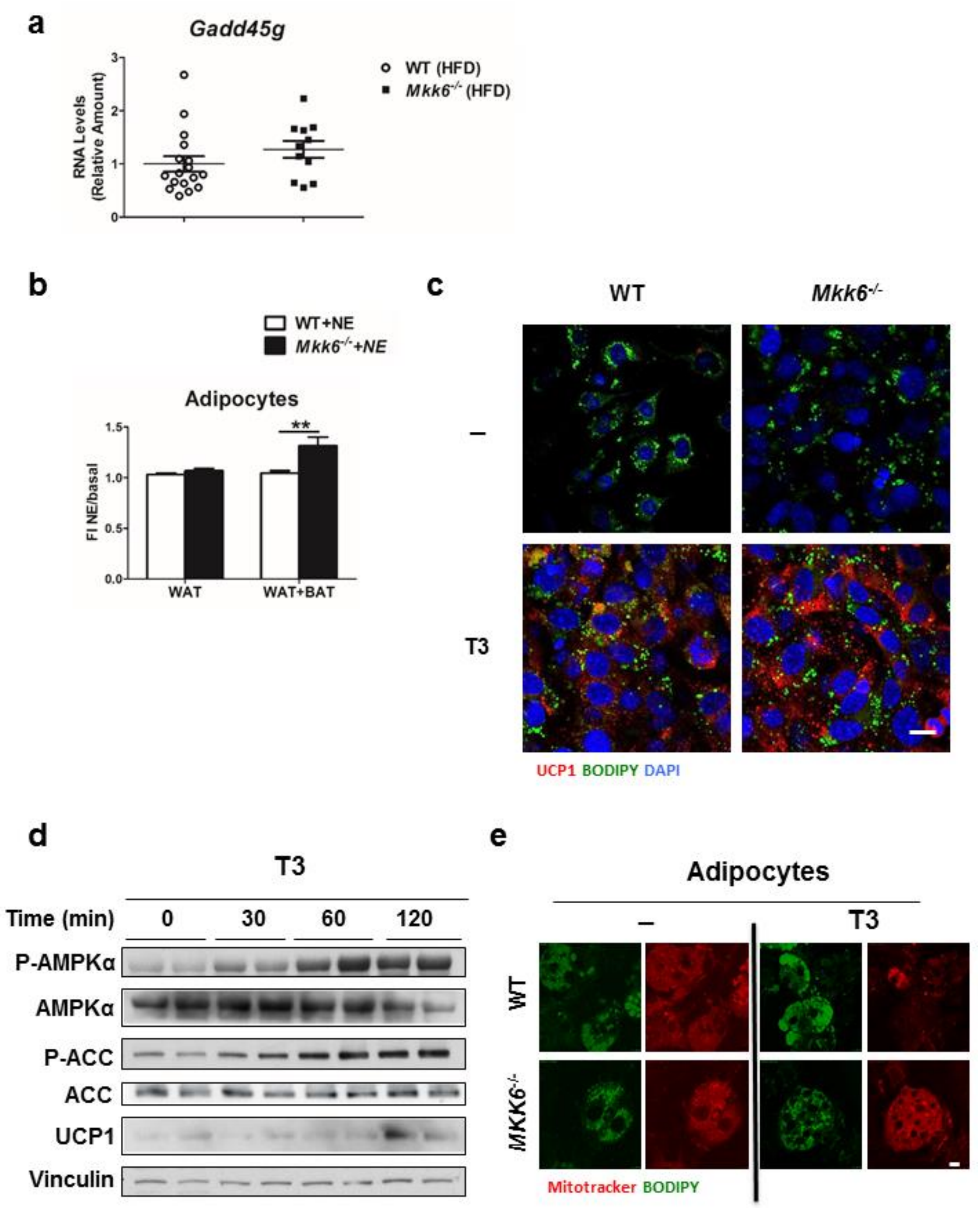

Supplementary Figure 5: Activation of WAT in *MKK6*$^{-/-}$ mice

(a) qRT-PCR analysis of *Gadd45g* in epididymal white fat (eWAT) from WT or *Mkk6*$^{-/-}$ mice fed a HFD. mRNA expression was normalized to the amount of *Gapdh* mRNA (mean±SEM, WT n=17 mice; *Mkk6*$^{-/-}$ n=11 mice). **(b)** Comparison of oxygen consumption response to norepinephrine (NE) in WAT adipocytes differentiated according to BAT adipocyte differentiation protocol (with prolonged exposure to T3) (WAT+BAT) versus normal protocol (WAT) by Seahorse assay. (mean±SEM, n=21 WT or 17 Mkk6-/- wells from 3 mice cultured separately) **(c)** Representative images of

T3-induced UCP1 expression in WT and *Mkk6*$^{-/-}$ adipocytes. Adipocytes were differentiated *in vitro* and stimulated 48 h with T3. UCP1 staining is shown in red, Bodipy in green (staining intracellular lipid accumulations), and DAPI in blue. Scale bar: 10 µm **(d)** Activation of AMPK pathway, as well as UCP1 expression, in eWAT from WT mice fed a ND treated injected with T3 (12 µg/100g in 0.2% BSA-PBS) during 30, 60 or 120 min n=4 mice . **(e)** Confocal imaging of T3-treated WT and *Mkk6*$^{-/-}$ primary adipocytes stained with mitotracker Deep Red (red) and Bodipy (green). $^{**}p < 0.01$ WT vs *Mkk6*$^{-/-}$ (Welch's test)

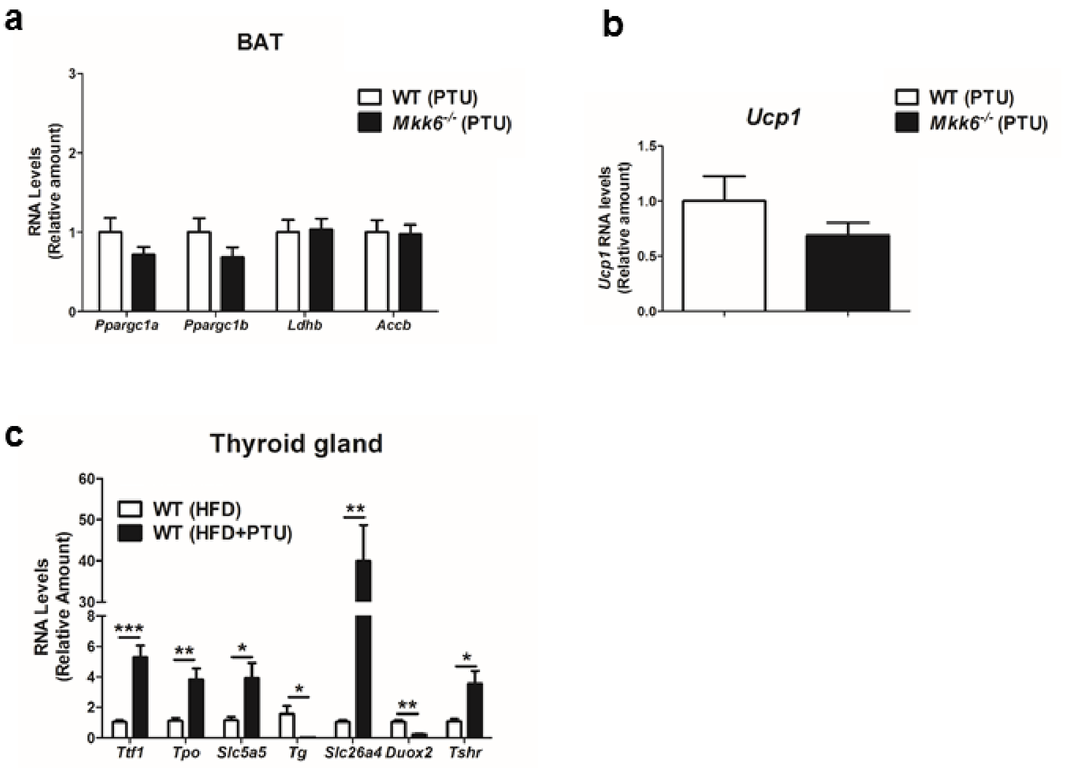

Supplementary Figure 6: Blockade of thyroid hormone synthesis does not affect BAT activity and suppresses thyroid hormone synthesis.

(a, b) qRT-PCR analysis of genes associated with brown adipose tissue (BAT) activity determined in total RNA extracted from BAT of WT and *Mkk6*$^{-/-}$ mice treated with propylthiouracil (PTU) during the 8-wk HFD period. mRNA expression was normalized to the amount of *Gapdh* mRNA. (mean±SEM, WT n=10 mice; *Mkk6*$^{-/-}$ n=9 mice) **(c)** To test PTU inhibition, qRT-PCR analysis of genes implicated in TH production in thyroid gland was assayed in fasted 8-wk HFD treated mice and in fed 8-wk HFD plus PTU treated mice. mRNA expression was normalized to the amount of *Hprt* mRNA. *Slc5a5* gene corresponds to the basal sodium (Na)-iodide symporter –Nis-, and *Slc26a4* gene to the apical iodine transporter Pendrin. All of these genes are implicated in the synthesis of thyroid hormones by the follicular cells of the thyroid. PTU blocks the activity of the enzyme thyroperoxidase (Tpo) leading to suppression of downstream genes (Thyroglobulin –Tg- and Dual-Oxidase 2 –Duox2-) and upregulation of its own expression (Tpo) and upstream genes (Ttf1 –Nkx2.1-, Nis, Pendrin and Tshr). Results are expressed as mean±SEM (HFD n=5 mice; HFD+PTU n=10 mice) *p < 0.05, **p < 0.01, ***p < 0.001, HFD vs HFD+PTU (Welch's test).

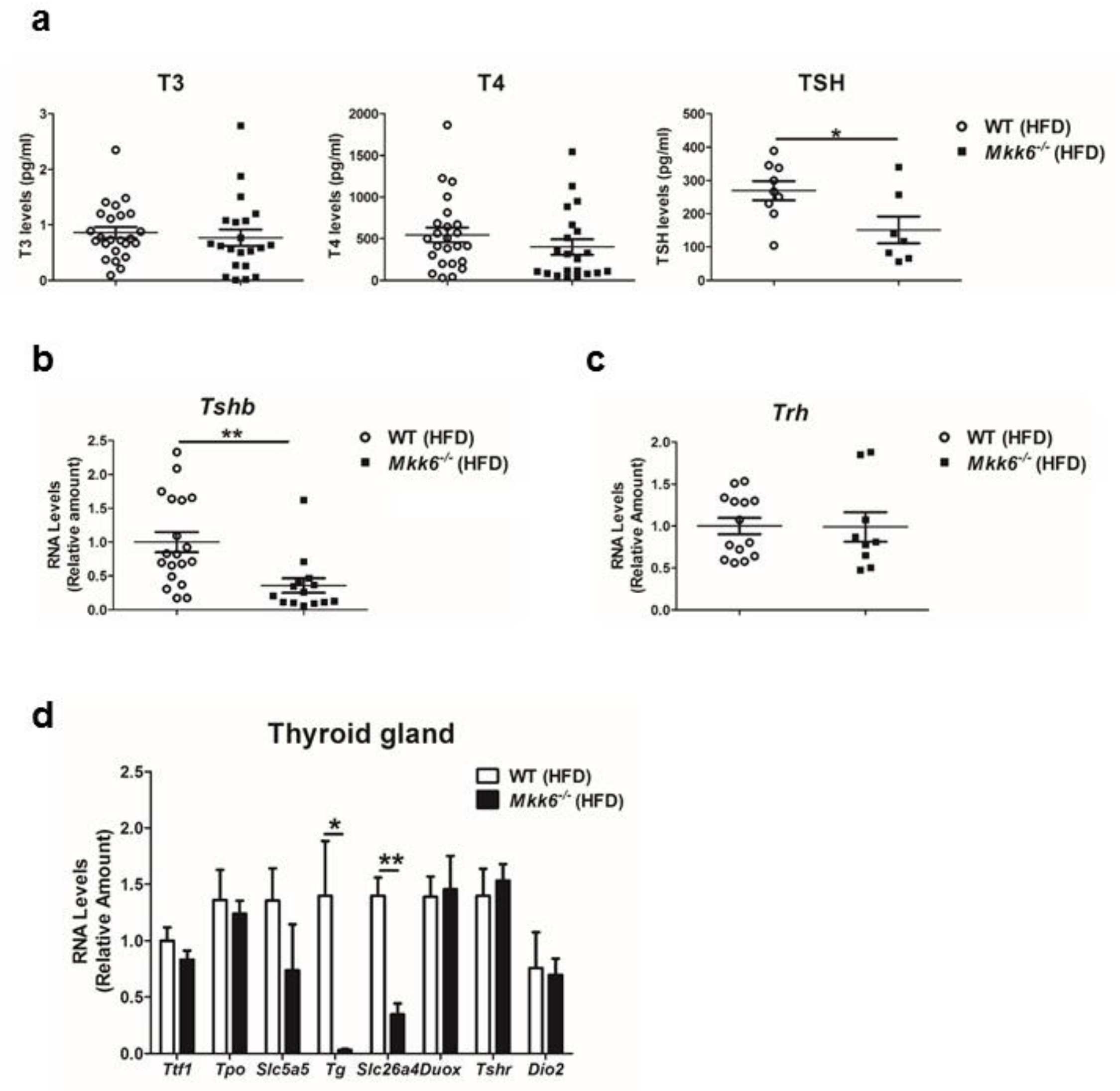

Supplementary Figure 7: Elevated thermogenesis in *Mkk6*$^{-/-}$ mice is not due to activation of Hypothalamic-Pituitary-Thyroid axis.

WT and *Mkk6*$^{-/-}$ mice were fed 8-wk HFD. **(a)** Circulating levels of T3, T4, and thyroid-stimulating hormone (TSH) (mean±SEM, T3, T4 WT n=24; *Mkk6*$^{-/-}$ n=21; TSH WT n=9 mice; *Mkk6*$^{-/-}$ n=7 mice). **(b, c)** qRT-PCR analysis of *Tshb* in pituitary gland and *Trh* (thyrotropin-releasing hormone) in total hypothalamic content was assayed. mRNA expression was normalized to the amount of *18s* or *Gapdh* mRNA, respectively. (mean±SEM, *Tsh* WT n=19 mice; *Mkk6*$^{-/-}$ n=14 mice; *Trh* WT n=14 mice; *Mkk6*$^{-/-}$ n=9 mice). **(d)** qRT-PCR analysis of genes implicated in TH production in thyroid gland. mRNA expression was normalized to the amount of *Hprt* mRNA. Supression of Tg and Pendrin (Slc26a4) in the thyroid together with *Tshb* expression and TSH hormone secretion by the pituitary indicates a hypersensitivity to the T3-dependent negative feedbacks. Results are expressed as mean±SEM (WT n=5 mice; *Mkk6*$^{-/-}$ n=3 mice). *$p < 0.05$, **$p < 0.01$, WT vs *Mkk6* $^{-/-}$ (*t*-test or Welch's test when variances were different).

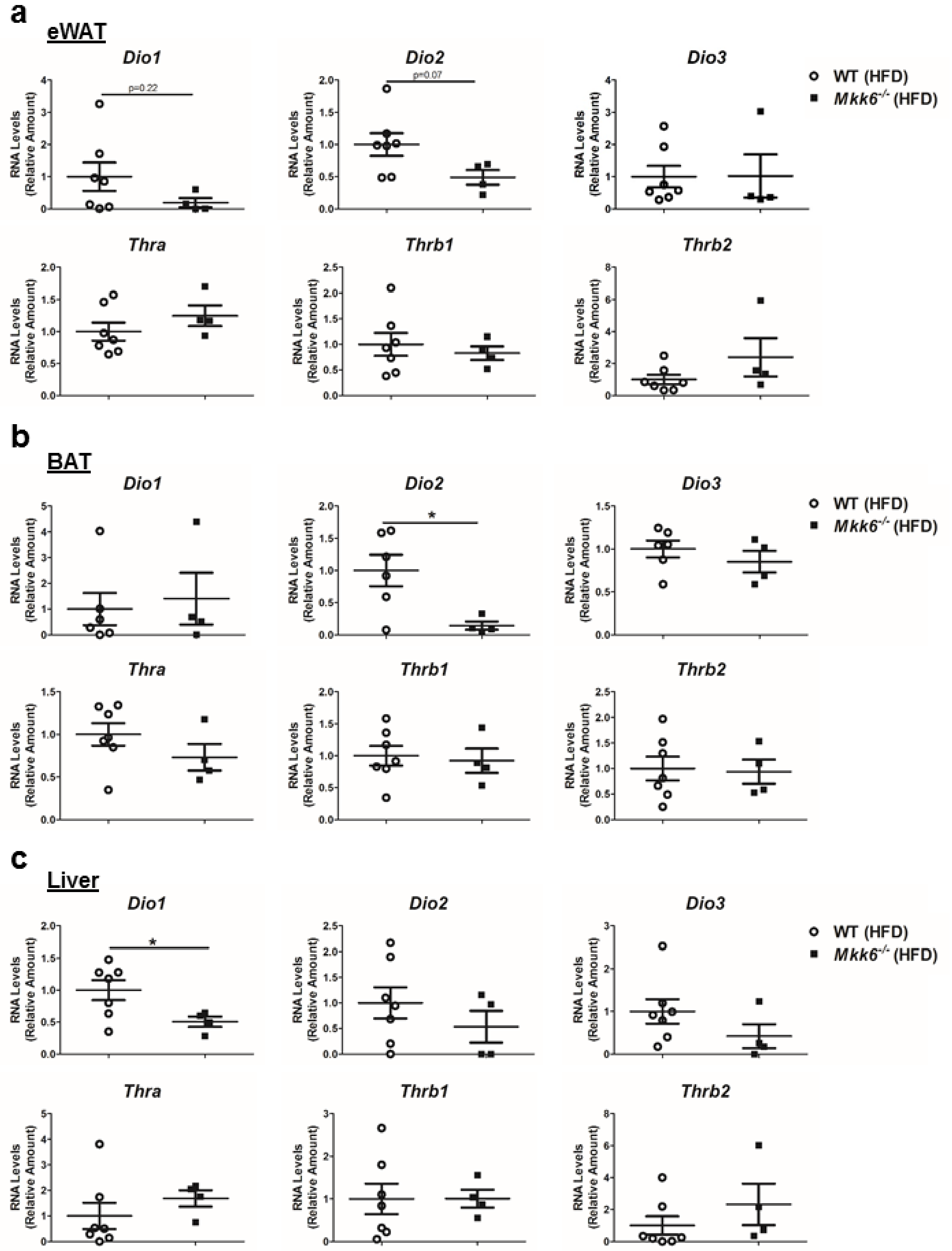

Supplementary Figure 8: TH receptors and Dio enzymes do not participate in *Mkk6*$^{-/-}$ mice phenotype.

WT and *Mkk6*$^{-/-}$ mice were fed 8-wk HFD. **(a, b, c)** qRT-PCR analysis of deiodinases (Dio1, 2, 3) and TH alpha and beta receptors in epidydimal white fat (eWAT) **(a)**, brown adipose tissue (BAT) **(b)**, and liver **(c)**. mRNA expression was normalized to the amount of *18s* or *Gapdh* mRNA, respectively. Results are expressed as mean±SEM (WT n=7 mice; *Mkk6*$^{-/-}$ n=4 mice). *p < 0.05, WT vs *Mkk6* $^{-/-}$ (*t*-test or Welch's test when variances were different).

a

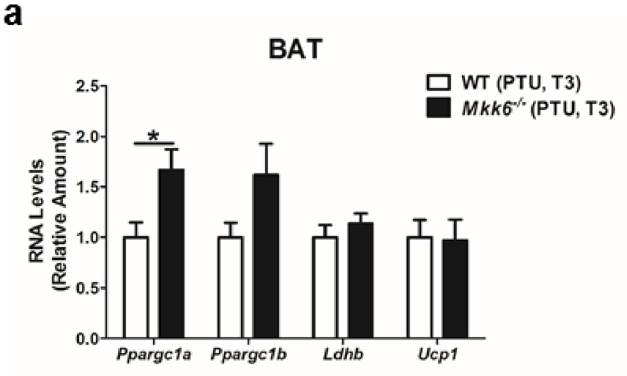

b

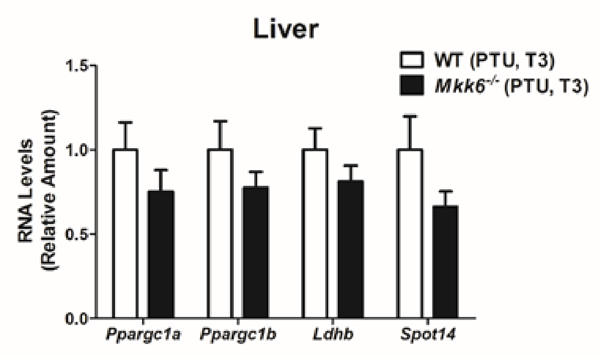

Supplementary Figure 9: Liver and brown adipose tissue do not show hypersensitivity to T3 administration.

WT and *Mkk6*$^{-/-}$ mice were treated with 1.2 mM propylthiouracil (PTU) during the 8-wk HFD period. After the first 2 weeks of treatment, mice received daily i.p. injections with T3 (3 µg/100g in 0.2% BSA-PBS). **(a, b)** qRT-PCR analysis of thyroid-hormone-responsive genes determined in total RNA extracted from brown adipose tissue (BAT) **(a)** or liver **(b)** of WT and *Mkk6*$^{-/-}$ mice after at the end of the treatment period. mRNA expression was normalized to the amount of *Gapdh* mRNA. Results are expressed as mean±SEM (WT n=9 mice; *Mkk6*$^{-/-}$ n=10 mice). *p < 0.05, WT vs *Mkk6*$^{-/-}$ (t-test).

Supplementary Figure 10: Uncropped Western blot images

Figure 1a

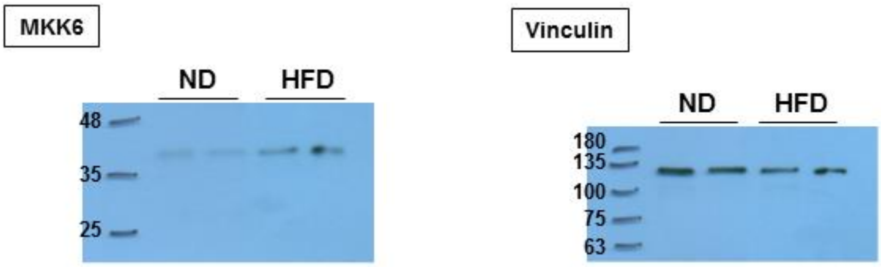

Figure 1j

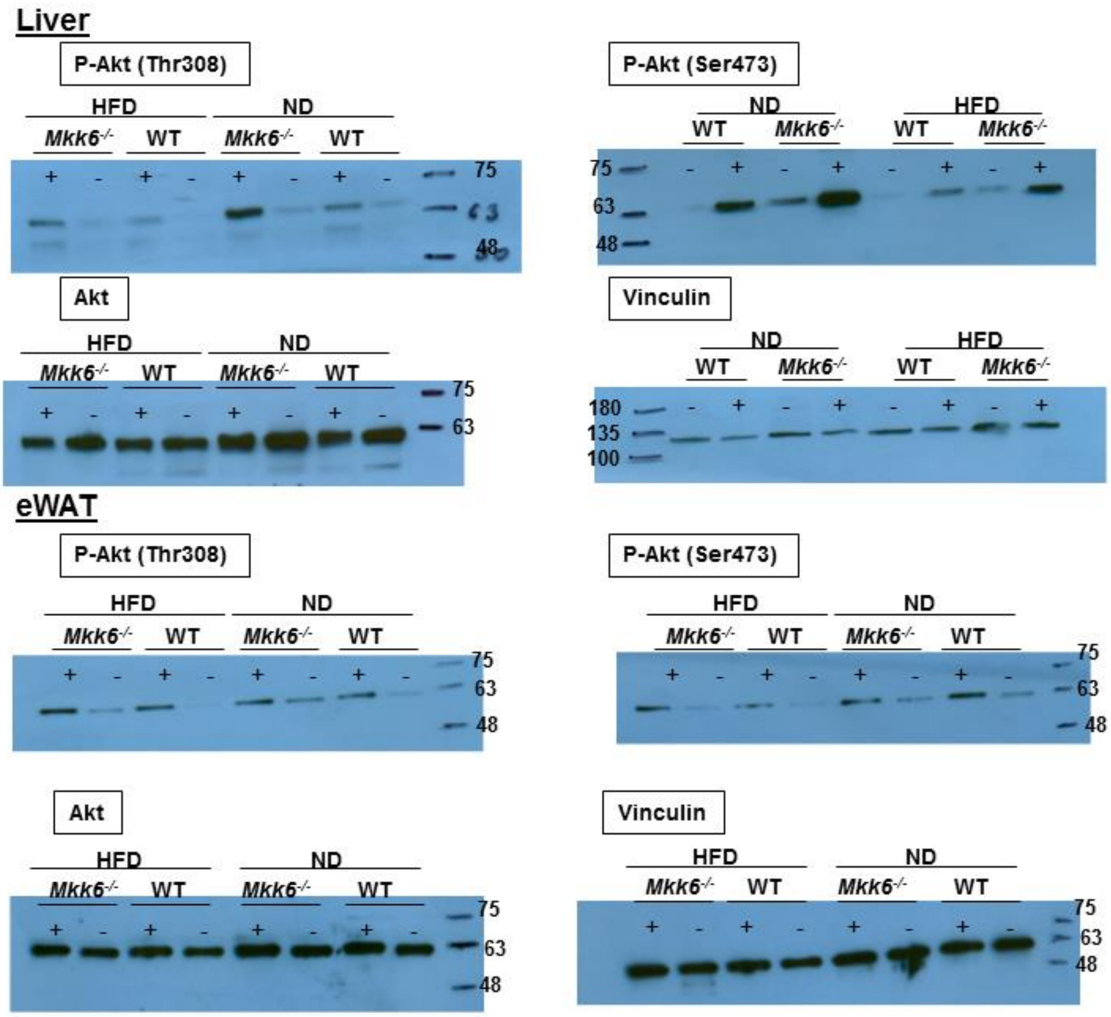

Supplementary Figure 10 (continued)

Figure 1j (continued)

Muscle

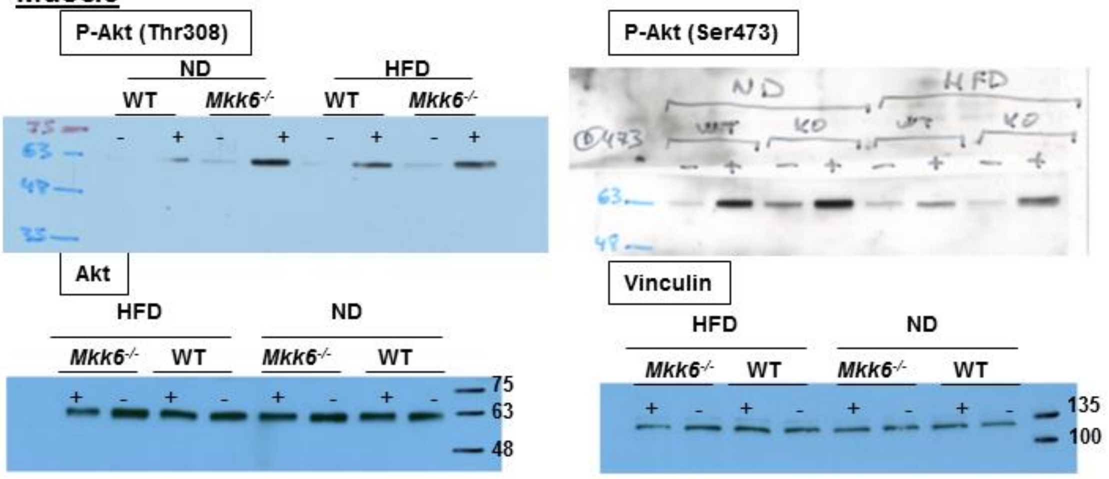

Figure 2e

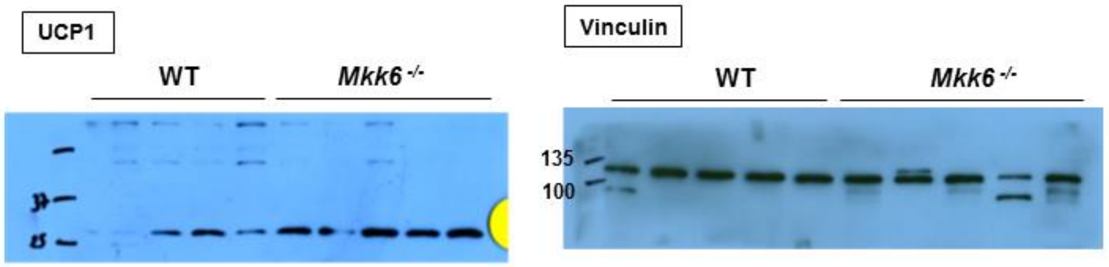

Figure 3a

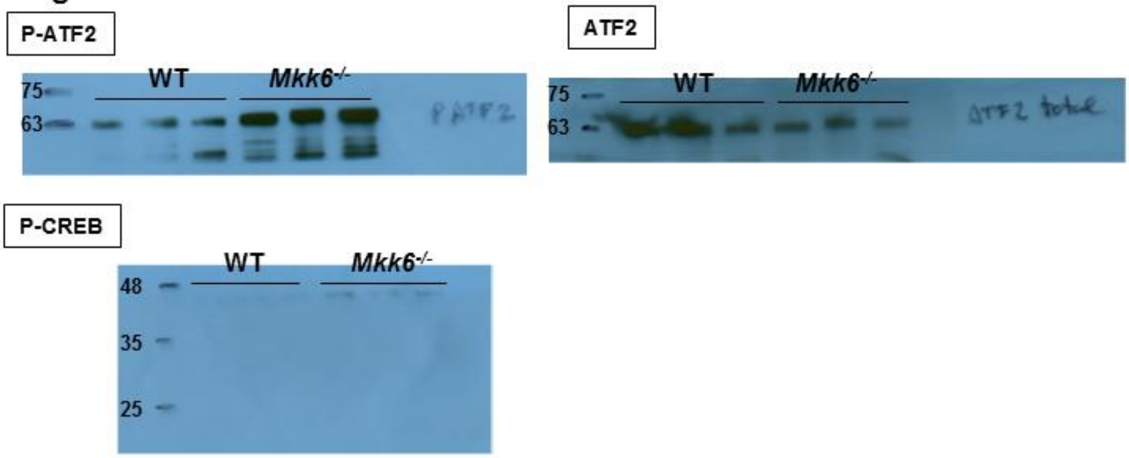

Supplementary Figure 10 (continued)

Figure 3a (continued)

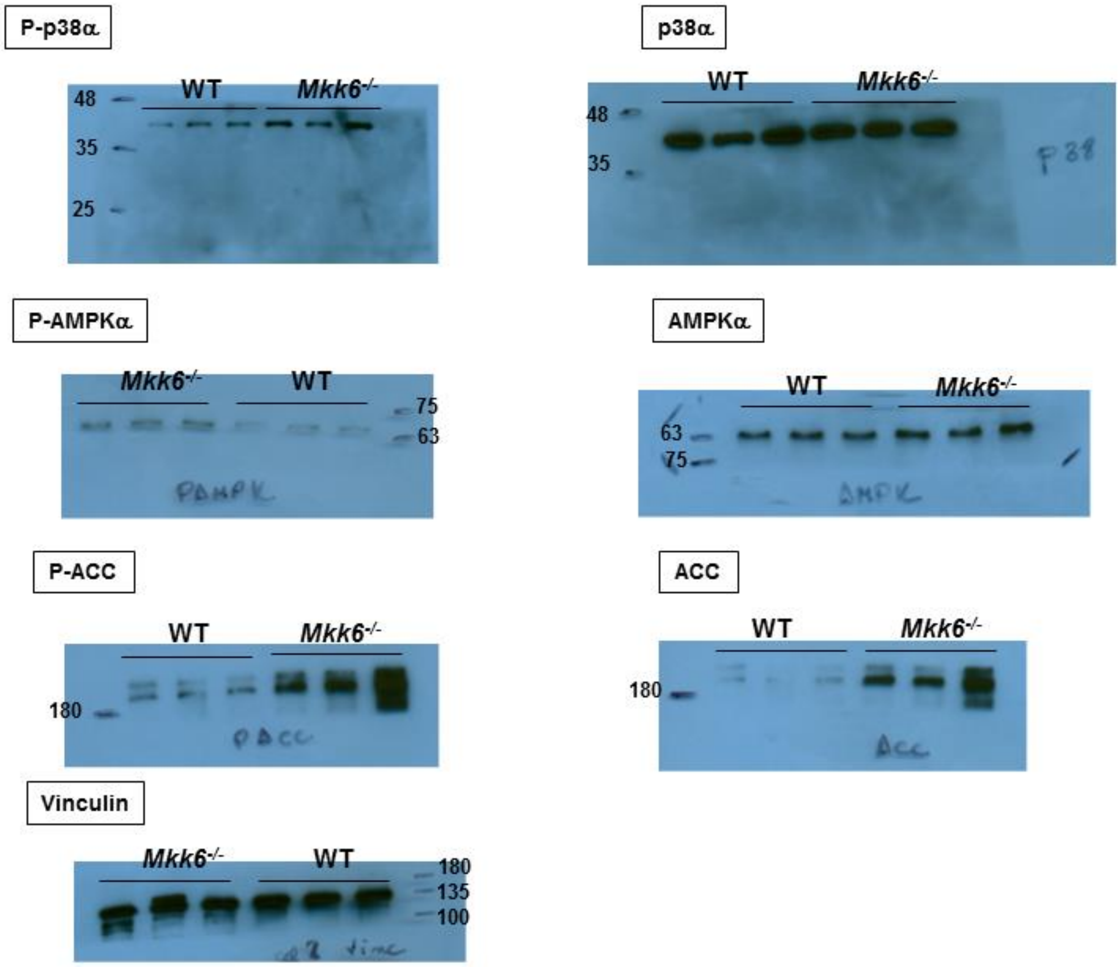

Figure 3b

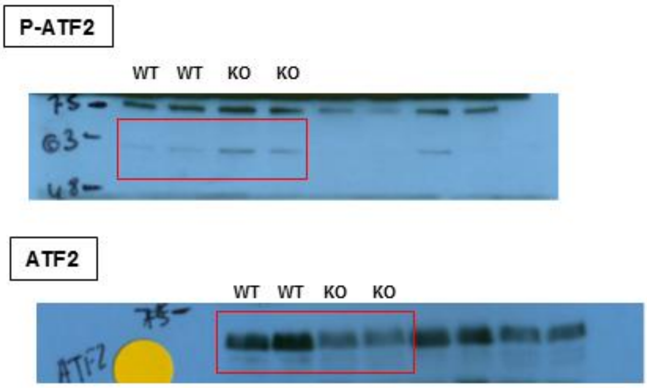

Supplementary Figure 10 (continued)

Figure 3b (continued)

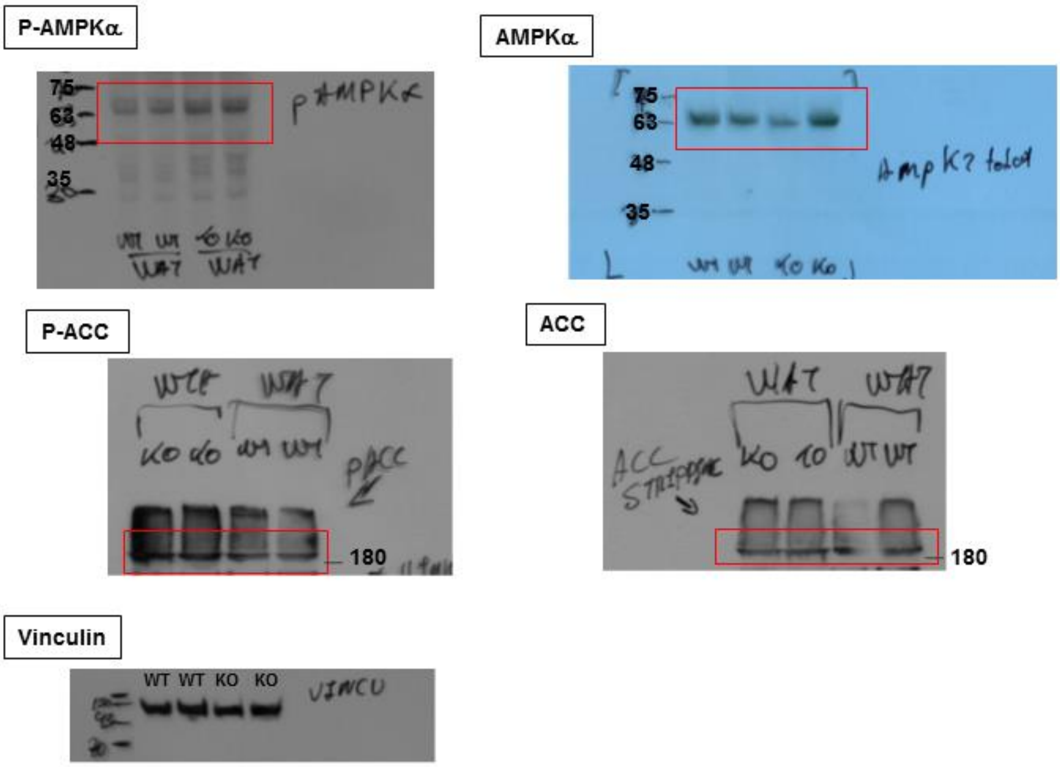

Figure 3c

AMPK

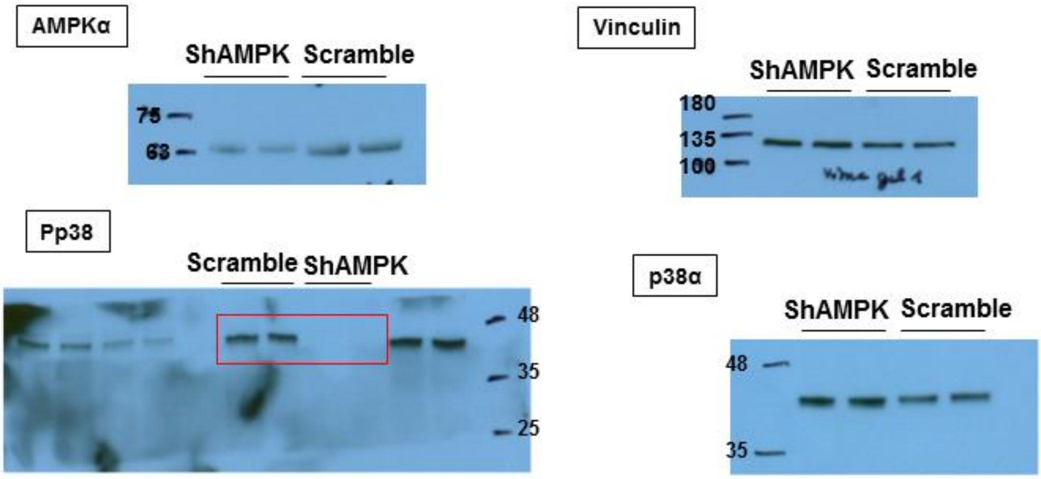

Supplementary Figure 10 (continued)

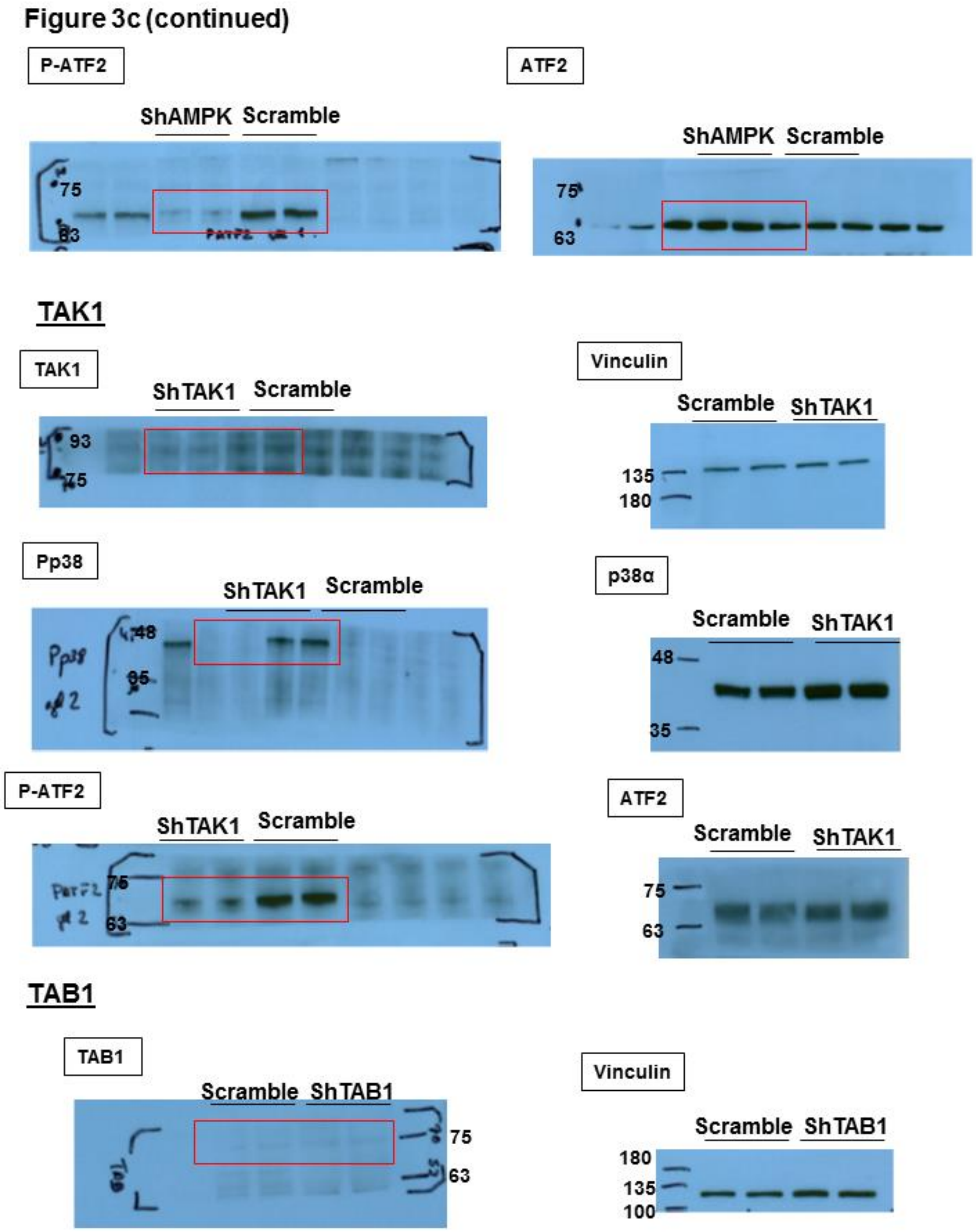

Supplementary Figure 10 (continued)

Figure 3c (continued)

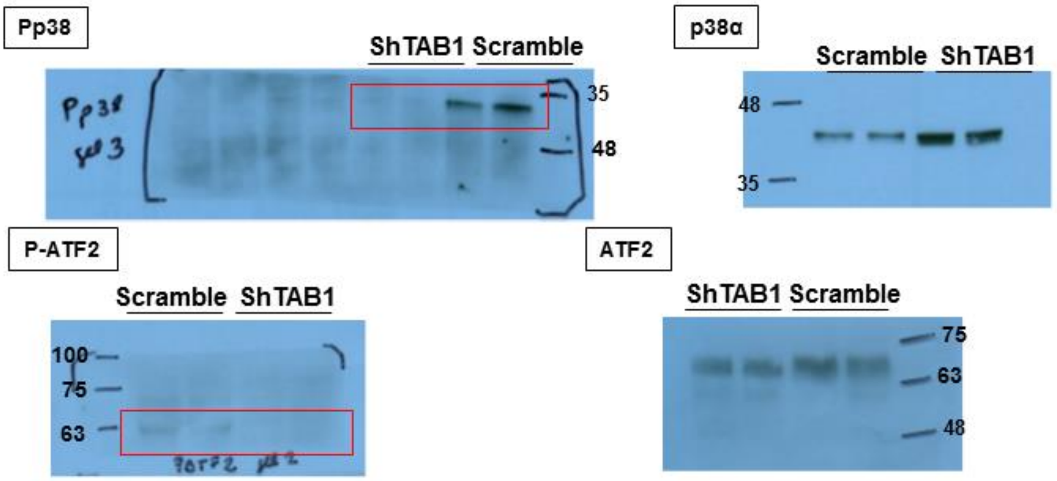

Figure 3d

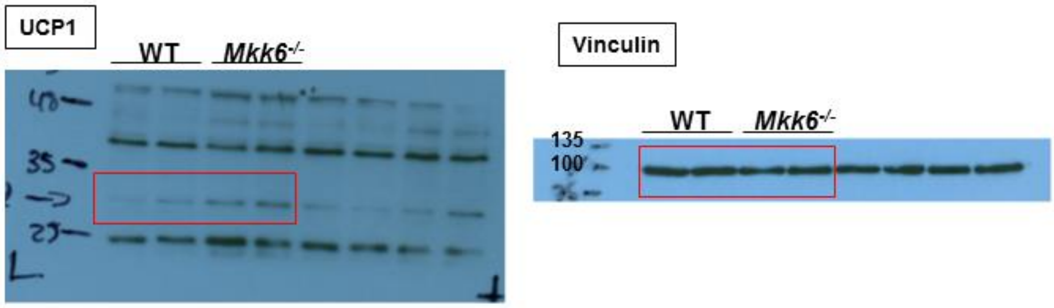

Figure 3e

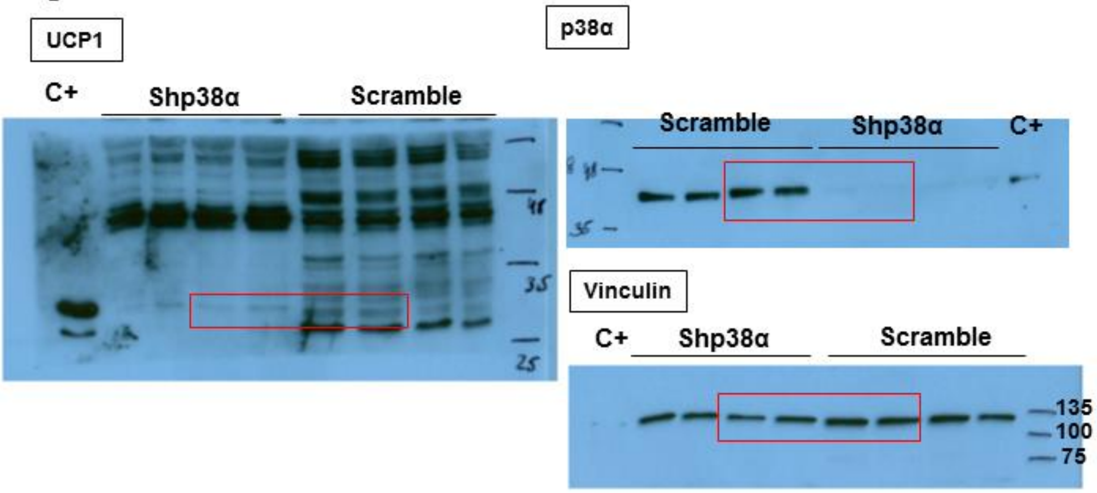

Supplementary Figure 10 (continued)

Figure 3h

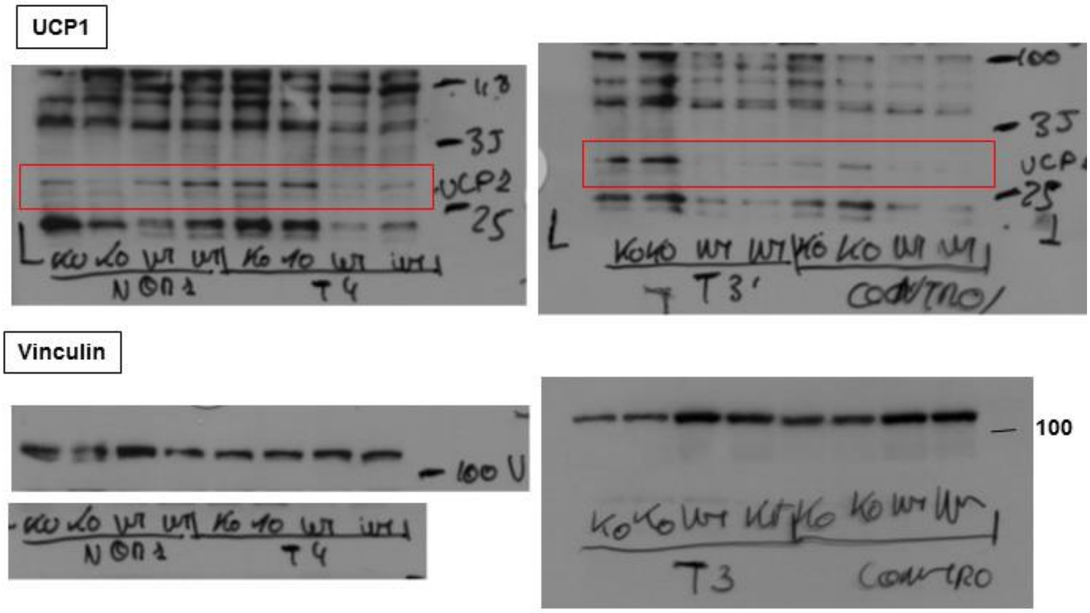

Figure 4g

eWAT

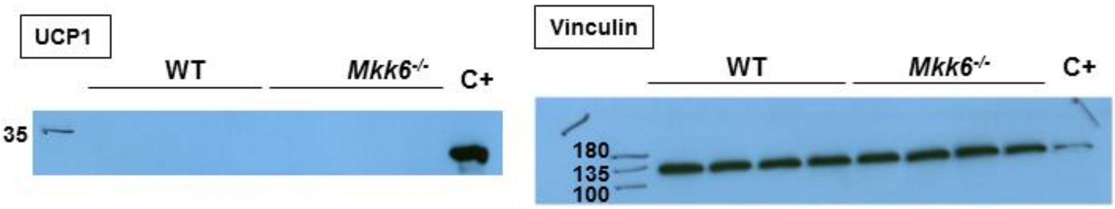

iWAT

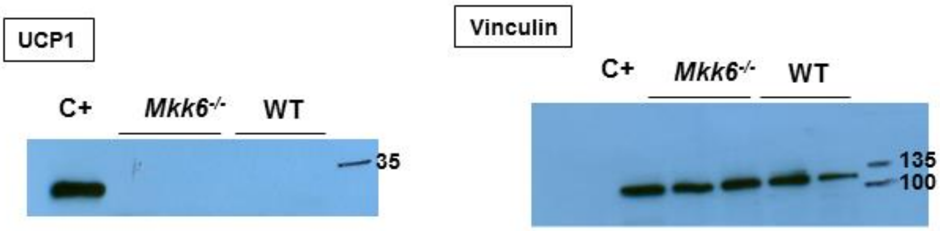

Supplementary Figure 10 (continued)

Figure 5g

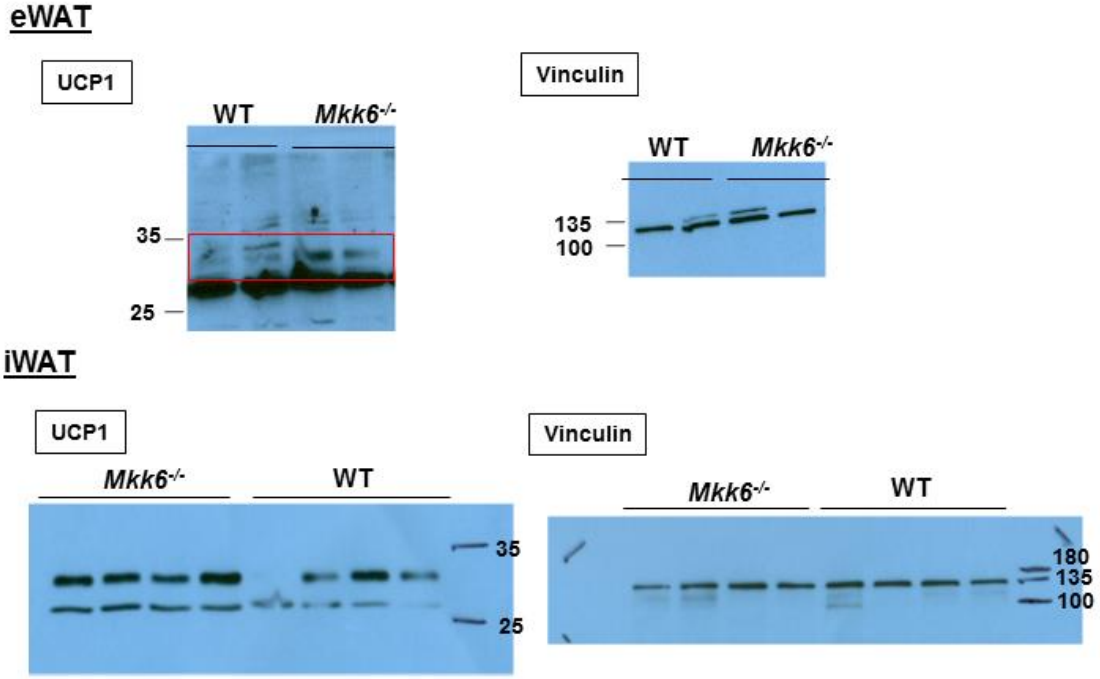

Figure 6e

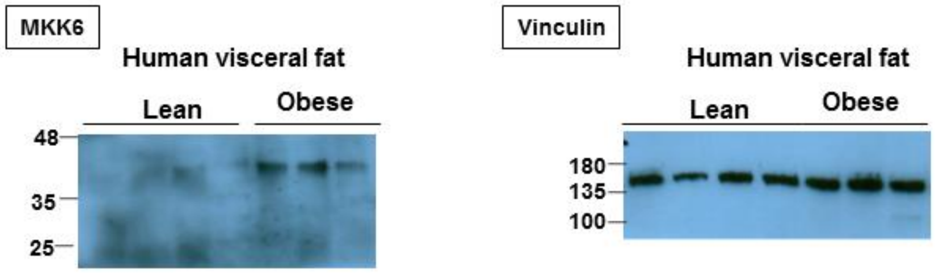

Supplementary Table 1. Characteristics of patients and controls

Variable	Obese patients (n = 53)	Controls (n = 18)	P
Age (years)	46.91 (12.37)	53.22 (15.85)	0.177
Female:male ratio	37:16	10:8	0.269
Hypertension (n)	26 (49.10)	6 (33.30)	0.322
Diabetes mellitus (n)	14 (26.40)	2 (11.10)	0.369
BMI (kg/m^2)	48.47 (6.87)	25.43 (3.47)	<0.0001
Fasting blood sugar (mg/dL)	113.34 (43.95)	94.06 (12.81)	0.050
AST (IU/L)	23.1 (12.43)	29.82 (27.03)	0.280
ALT (IU/L)	30.5 (17.92)	70.06 (162.67)	0.791
Alkaline phosphatase	75.08 (27.17)	91.75 (35.63)	0.072
Bilirubin (mg/dL)	0.42 (0.13)	0.63 (0.32)	0.011
Albumin (mg/dL)	4.27 (0.25)	4.45 (0.45)	0.010
Total cholesterol (mg/dL)	188.96 (32.78)	202.69 (41.96)	0.162
Triglycerides (mg/dL)	145.33 (71.62)	136.7 (49.78)	0.931
LDL-cholesterol (mg/dL)	111.37 (33.03)	124.8 (38.69)	0.276
HDL-cholesterol (mg/dL)	46.61 (12.57)	50.63 (18.83)	0.863

Variables are presented as mean (standard deviation) or absolute frequency (percentage) and are compared by means of Mann-Whitney U test or χ^2 test. BMI: body mass index. AST: aspartate aminotransferase. ALT: alanine aminotransferase.

Supplementary Table2. Primers and probes used for gene amplification.

Gene	Forward	Reverse
Gapdh	TGAAGCAGGCATCTGAGGG	CGAAGGTGGAAGAGTGGGA
Ppargc1a	TATGGAGTGACATAGAGTGTGCT	CCACTTCAATCCACCCAGA
Ppargc1b	GCTCTGACGCTCTGAAGGAC	AAGGGCTTGGGCAATCCTC
Ldhb	AGCTCAAAGGAGAGATGATG	GAATGATGAACTTGAACACC
Accb	CCTTTGGCAACAAGCAAGGTA	AGTCGTACACATAGGTGGTCC
Ucp1	GTGAACCCGACAACTTCCGAA	TGAAACTCCGGCTGAGAAGAT
Cidea	TGACATTCATGGGATTGCAGAC	GGCCAGTTGTGATGACTAAGAC
Cpt2	CAGCACAGCATCGTACCCA	TCCCAATGCCGTTCTCAAAAT
Cox7a2	GCTGGCCCTTCGTCAGATT	GGCATCCCATTATCCTCCTGAA
Cox8b	TGTGGGGATCTCAGCCATAGT	AGTGGGCTAAGACCCATCCTG
Prdm16	CCACCAGCGAGGACTTCAC	GGAGGACTCTCGTAGCTCGAA
Cox7a1	GCTCTGGTCCGGTCTTTTAGC	GTACTGGGAGGTCATTGTCGG
Cpt1a	CTCCGCCTGAGCCATGAAG	CACCAGTGATGATGCCATTCT
Cpt1b	GCACACCAGGCAGTAGCTTT	CAGGAGTTGATTCCAGACAGGTA
Gadd45g	GGGAAAGCACTGCACGAACT	AGCACGCAAAAGGTCACATTG
Trh	ACATGCCAGGAAGGCGTTT	TCCAAAGCATCATTAGGCTGAAA
Thra	CTGACCTCCGCATGATCGG	GGTGGGGCACTCGACTTTC
Thrb1	ACACCAGCAATTACCAGAGTG	GCAGCTCGAAGGGACATGA
Thrb2	CCAGAGGTACACGAAGTGTGC	AGGTTTCCAGGGTAACTACAGG
Spot14	ATGCAAGTGCTAACGAAACGC	GGAGTACCGATCCATGACTGTC
Tshr	GACCCTGAAACTGTACAACAATGG	CCTCCAAAGGCATCGTTGTC
Tpo	CCGCTGCTGAGAAAAGGAAA	GCATTATTGCCATAGCTCCAAGTG
Tg	GCTGAGACAGGACTGGAATTGTT	ATATCCGGTACATGGTGCTTTGAG
Duox2	CCACCATGCTGTACATCTGTGA	AGGGAGGGCGACCAAAGT
Slc5a5	GCTGCTCACAGCCTTGCTCTT	GCGCAGTTCTAGGTACTGGTAGGT
Slc26a4	TGACATCATCTCCGGAGTTAGCA	GGCATAAGCCATCCCTTGCA
18s	430448607052 Applied Bios, ThermoFisher	430449007049, Applied Bios, ThermoFisher
Tsh	Mm00437190_m1, Applied Biosystems, ThermoFisher	
Dio1	Mm00839358_m1, Applied Biosystems, ThermoFisher	
Dio2	Mm00515664_m1, Applied Biosystems, ThermoFisher	
Dio3	Mm01325881_s1, Applied Biosystems, ThermoFisher	
Ttf1	Hs00163037_m1, Applied Biosystems, ThermoFisher	
Hprt	AGCCGACCGGTCCCGTCAT	GGTCATAACCTGGTTCATCATCGC<br>Pb:<br>CGACCCGCAGTCCCAGCGTCGTGAT
